# Quorum sensing employs a dual regulatory mechanism to repress T3SS gene expression

**DOI:** 10.1128/mbio.00106-25

**Published:** 2025-02-25

**Authors:** Payel Paul, Ram Podicheti, Logan J. Geyman, Elizabeth N. Baker, Kai Papenfort, Douglas B. Rusch, Julia C. van Kessel

**Affiliations:** 1 Department of Biology, Indiana University Bloomington 123993 , Bloomington, Indiana, USA; 2 Center for Genomics and Bioinformatics, Indiana University 151786 , Bloomington, Indiana, USA; 3 School of Medicine, Indiana University, Indianapolis, Indiana, USA; 4 Institute of Microbiology, Friedrich Schiller University, Jena, Thuringia, Germany; 5 Microverse Cluster, Friedrich Schiller University Jena 9378 , Jena, Thuringia, Germany; University of Illinois Chicago, Chicago, Illinois, USA

**Keywords:** quorum sensing, *Vibrio campbellii*, *Vibrio*, type III secretion, T3SS

## Abstract

The type III secretion system (T3SS) is a needle-like complex used by numerous bacterial pathogens in host infection to inject exotoxins into the host cell cytoplasm. The T3SS is a known virulence factor in the shrimp pathogen *Vibrio campbellii*. The ~40 genes comprising the *V. campbellii* T3SS are regulated by a network of transcription factors in response to changes in the cell’s environment: cell density (quorum sensing; QS), temperature, calcium, and host cell contact. Under positive environmental stimuli, the master T3SS transcription factor ExsA activates the expression of the four structural T3SS operons required for needle formation. Previous studies identified a key role of the master QS transcription factor LuxR: repression of *exsA* transcription via DNA binding at the *exsBA* promoter. Here, we uncovered a new regulatory role of LuxR: post-translational repression of ExsA activity via transcriptional repression of the gene encoding the anti-anti-activator ExsC. In *V. campbellii*, ExsC is a positive regulator of T3SS transcription; deletion of *exsC* decreases ExsA-dependent transcription activation of the T3SS structural promoters. Through genetic epistasis and *in vitro* biochemical assays, we show that LuxR directly binds the *exsC* promoter upstream of ExsA and represses transcription of *exsC*. Our findings collectively show that *V. campbellii* responds to high cell density signals to shut down ExsA-dependent expression of the T3SS via two mechanisms. We postulate that this dual regulatory mechanism by LuxR enables both the rapid inactivation of existing ExsA protein and blocks its further synthesis, leading to a rapid shutdown of T3SS activity at high cell density.

**IMPORTANCE:**

*Vibrio campbellii* utilizes the type III secretion system (T3SS) as a mechanism of pathogenesis, which is a highly studied “injectisome” complex that delivers exotoxins into host cells during infection. The T3SS pathogenicity island in *V. campbellii* comprises ~40 genes that are organized into four structural operons. In this study, we determined that quorum sensing—a method of bacterial communication—regulates T3SS genes both at the transcriptional and post-translational levels to shut down T3SS gene expression at high population densities.

## INTRODUCTION

*Vibrio campbellii* is a gram-negative γ-proteobacterium that belongs to the class *Vibrionaceae*, many of which are recognized as pathogens of aquatic animals, including shrimp, lobster, mollusk, and shellfish. (1–5). Pathogenic strains of *V. campbellii* are known to cause diseases in shrimp, such as acute hepatopancreatic necrosis disease (AHPND), red spot syndrome, white tail disease, and luminous vibriosis (1, 3, 6–8). In our study, we use *V. campbellii* BB120 (*a*lso known as ATCC BAA-1116, previously classified as *V. harveyi*) (9) as a model organism to study the mechanisms of virulence in pathogenic strains of *V. campbellii*.

A major mechanism of virulence in gram-negative pathogens is the type III secretion system (T3SS) machinery, a specialized secretion apparatus which plays an essential role in secretion of extracellular protein toxins. It is a syringe-like membrane-embedded apparatus also called the “injectisome,” which contacts the eukaryotic host cell membrane and injects effector proteins or exotoxins across the membrane and into the host cell (10, 11). Exotoxins compromise the host cell machinery and activate cell lysis through various mechanisms that have been broadly studied in pathogens including *Yersinia pestis*, *Pseudomonas aeruginosa*, *Salmonella typhimurium*, and *Vibrio parahaemolyticus* (12–17). Certain species of the *Vibrionaceae* family such as *V. parahaemolyticus* possess two types of T3SSs encoded by entirely separate sets of structural genes and carrying distinct arsenals of effector proteins (12, 13, 18). The two T3SSs are broadly differentiated based on the target and general mechanism of action of the effector proteins (12, 13, 18).

Previous studies identified a functional T3SS in *V. campbellii* BB120 and determined that QS negatively regulates T3SS at high population densities (19–21). Furthermore, QS-mediated regulation of T3SS has been shown to be an important virulence factor in *V. campbellii* infection in shrimp (22). All these published data thus far indicate that the T3SS of *V. campbellii* BB120 is similar in structure, function, and genetic organization to the T3SS1 of *V. parahaemolyticus; V. campbellii* BB120 does not encode a T3SS2 (19, 21). Thus, we focus here on T3SS1 function and regulation in *V. campbellii* and will refer to this system in this manuscript simply as “T3SS.”

**Fig 1:**
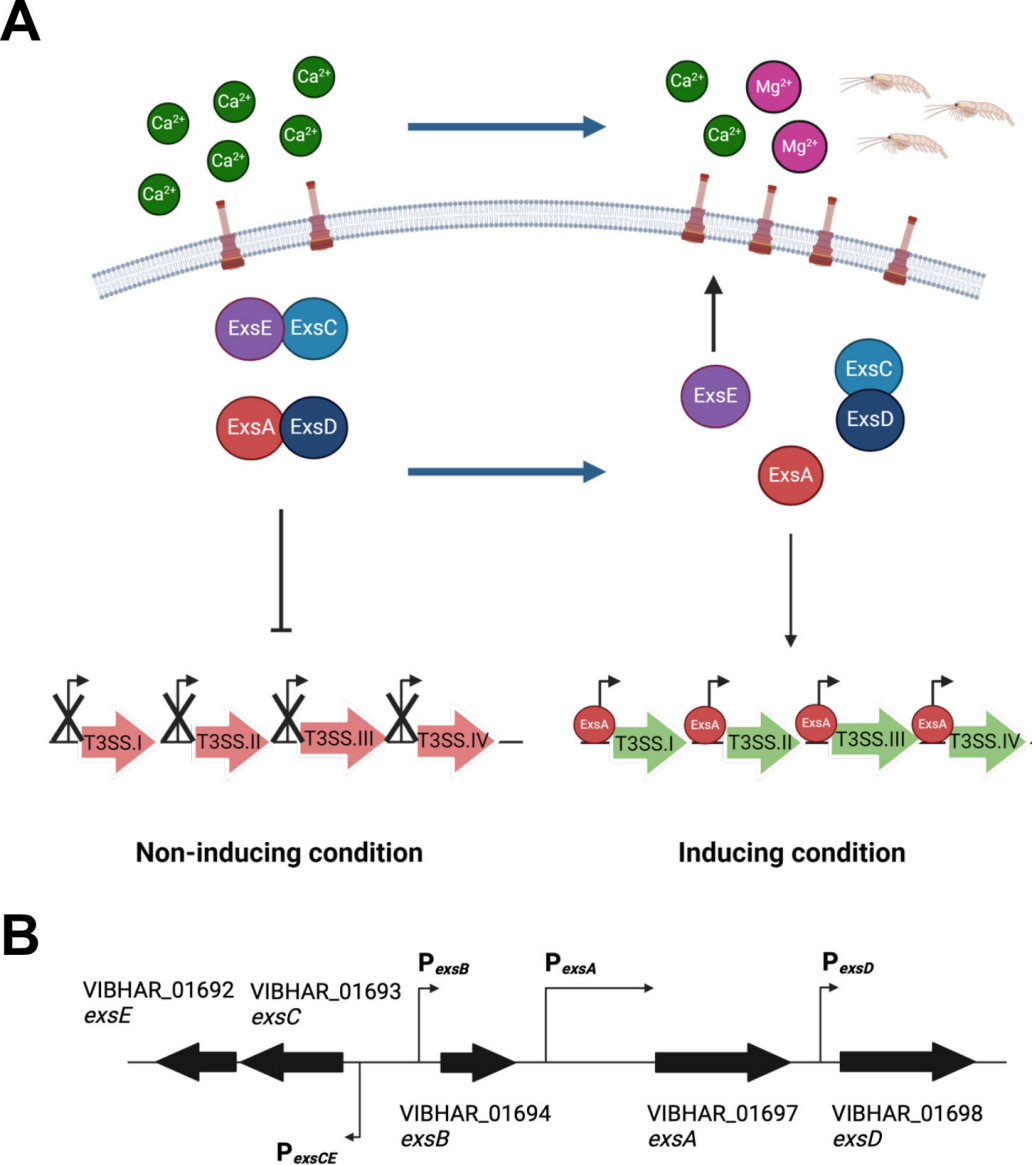
Schematic models of the Exs regulatory cascade and the organization of the *exs* regulatory genes in *V. campbellii*. (**A**) Schematic model of the regulatory networks that control T3SS gene expression in other organisms. The diagram shows the current model in the T3SS field in which secretion is induced upon T3SS contact with the host cell membrane during infection or upon chelation of calcium ions from media along with the addition of magnesium. The schematic shows the internal Exs regulatory cascade determined in *P. aeruginosa and V. parahaemolyticus*, which comprises ExsA, ExsD, ExsC, and ExsE. In the absence of induction, transcriptional regulator ExsA is bound to anti-activator ExsD, and anti-anti-activator ExsC is bound to ExsE. Upon induction of the system, ExsE is exported, freeing up ExsC to bind to ExsD, and ExsA protein is free to bind to T3SS promoters I–IV to activate transcription. (**B**) The schematic shows gene organization of T3SS regulatory genes *exsA*, *exsB*, *exsC*, *exsE,* and *exsD* in *V. campbellii* BB120 and their corresponding VIBHAR_XXXXX locus tags.

Bacterial pathogens regulate the expression of their virulence traits—such as T3SS—in response to external signals to initiate colonization of target tissues, avoid host immune system mechanisms, and persist. In *V. parahaemolyticus and P. aeruginosa,* the Exs proteins ExsA, ExsD, ExsC, and ExsE establish a regulatory cascade that dictates the activity of the T3SS1 master transcription activator ExsA (23–29) (Fig. 1A). In non-inducing conditions, *i.e*., the absence of a host cell membrane, or in media containing high levels of calcium and minimal magnesium, ExsA is bound by its anti-activator ExsD, and the T3SS genes are not transcribed by ExsA. Also in this condition, chaperone ExsC binds secretory protein ExsE. ExsE is not secreted while T3SS channels remain closed. Upon sensing inducing conditions, *i.e*., upon contact with a host cell membrane or the absence/decrease of calcium and addition of magnesium, T3 secretion is induced, allowing ExsE to be secreted by the T3SS (Fig. 1A). Once ExsE is secreted, ExsC is free to bind its other binding partner ExsD, which relieves the repression of ExsA. ExsA is then free to bind the downstream T3SS promoters and activate their transcription (27, 28, 30). In addition to these four factors, the *exsB* gene clusters with the regulatory *exs* genes. Gene *exsB* encodes a pilotin that attaches to the outer membrane and regulates the assembly of the type III secretin on the outer membrane (31). Collectively, the Exs regulatory cascade composes the internal post-translational regulatory system.

Previous studies of QS regulation showed that LuxR inhibits T3SS gene expression at high cell density through the direct binding of LuxR to the *exsB* and *exsA* promoters that drive the expression of *exsA*, resulting in no production of the T3SS activator ExsA (Fig. 1B) (20, 21). Further, LuxR primarily regulates *exsA* transcription through the *exsB* promoter; *exsA* and *exsB* are expressed as co-transcripts in *V. campbellii* (Fig. 1B) (21). In this study, we show evidence that LuxR binds and regulates transcription from both the *exsB* and *exsC* promoters in *V. campbellii* BB120, resulting in a dual mechanism of regulation: i) LuxR represses *exsA* transcription via the *exsB* promoter (21), and ii) LuxR regulates ExsA activity post-translationally through repression of *exsC* transcription. We hypothesize that the dual regulation allows the QS system to shut down the T3SS quickly and efficiently at high cell density.

## RESULTS

### A reporter assay for measuring T3SS regulation

**Fig 2:**
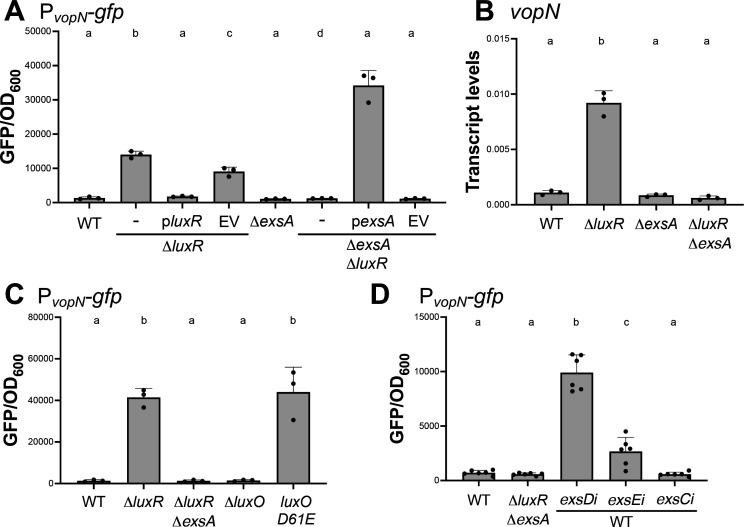
T3SS gene expression can be measured using a P*vopN-gfp* reporter. (**A**) GFP reporter assay results are shown for isogenic *V. campbellii* BB120 strains wild-type (WT), Δ*exsA*, Δ*luxR*, and Δ*exsA* Δ*luxR* containing the P*_vopN-gfp_* reporter (pPP26) and either no additional plasmid (–), the P*_tactheo_-exsA* expression plasmid (p*exsA*, pPP25) induced with 10 µM IPTG and 1 mM theophylline, the P*_tactheo_-luxR* expression plasmid (p*luxR*, pPP60) induced with 1 mM IPTG and 1 mM theophylline, or an empty vector control plasmid (EV). (**B**) RT-qPCR measurements of *vopN* transcripts from cells collected at HCD (OD_600_ = 1.0) compared to the internal control *hfq* gene. The strains assayed were wild-type (WT), Δ*exsA*, Δ*luxR*, and Δ*exsA* Δ*luxR*. (**C**) GFP reporter assay results are shown for isogenic *V. campbellii* BB120 strains wild-type (WT), Δ*luxR*, Δ*exsA* Δ*luxR*, Δ*luxO*, and *luxO D61E* containing the P*_vopN-gfp_* reporter. (**D**) GFP reporter assay results are shown for isogenic *V. campbellii* BB120 strains wild-type (WT) or Δ*luxR*Δ*exsA* containing the P*_vopN-gfp_* reporter. CRISPRi was used to knockdown gene expression in wild-type strains containing plasmids with guide RNAs targeting *exsD* (*exsDi*), *exsE* (*exsEi*), and *exsC* (*exsCi*) via induction with 100 µM IPTG. For panels A–C, the error bars represent the standard deviation of the mean. A one-way analysis of variance (ANOVA) test was performed on normally distributed data (Shapiro–Wilk test), followed by Tukey’s multiple comparisons test. Different letters indicate significant differences between strains in pairwise comparisons (*P* < 0.05; *n* = 3). For panel D, the error bars represent the standard deviation of the mean. A one-way ANOVA test was performed on normally distributed data (Shapiro–Wilk test), followed by Tukey’s multiple comparisons test. Different letters indicate significant differences between strains in pairwise comparisons (*P* < 0.05; *n* = 6).

To formally assess the predicted role of the T3SS regulatory network in our hands, we designed a reporter system to enable rapid quantification of T3SS gene expression. We constructed a reporter with a promoter-less *gfp* cassette fused to the promoter of *vopN*, which is also the promoter of the T3SS.II structural operon (Fig. S3A). The reporter operon was integrated into the chromosome of the *V. campbellii* strain using Tn7 transposition (32). We chose *vopN* because it is a structural gene regulated by ExsA but with no known role in regulating *exsA* expression, ExsA activity, or expression of other T3SS genes. As expected from previous studies, LuxR repressed *vopN* expression, and ExsA was required for activation of expression (Fig. 2A). These differences in *vopN-gfp* expression were not due to differences in growth rates (Fig. S2). Complementation of *luxR* under control of an autonomous promoter (P*_tactheo_-luxR*) restored the reporter phenotype to wild-type. Complementation of *exsA* under P*_tactheo_* in a Δ*exsA* Δ*luxR* background activated expression of P*_vopN_* to higher levels than the Δ*luxR* strain (Fig. 2A). The P*_vopN_* reporter results were corroborated with RT-qPCR (Fig. 2B). Of note, all assays measuring T3SS expression were induced in liquid media with 15 mM MgSO_4_ to obtain more robust expression of T3SS genes, as described in a previous study (33).

We next tested the expression of the *vopN* reporter in *V. campbellii* BB120 with genetically locked mutant strains that mimic the low cell density (LCD) and high cell density (HCD) conditions of QS. In *Vibrios*, the QS cascade involves multiple membrane-bound, histidine-kinase receptor proteins (Fig. S1) (34). In the absence of autoinducers at LCD, phosphorylation of the phospho-transfer protein LuxU by these receptor kinases (unbound by autoinducers) then phosphorylates the response regulator LuxO, which drives transcription of the Qrr regulatory RNAs and leads to LCD gene expression by AphA (35). Conversely, at HCD, autoinducer binding to the membrane-bound receptors reverses the phosphorylation cascade, resulting in unphosphorylated LuxO protein, no Qrr expression, and HCD gene expression by LuxR (34, 35). Thus, we used genetically locked mutant strains to mimic the LCD and HCD states: *luxO* D61E and Δ*luxO* strains, respectively (36–40). Of note, it was discovered that previous annotations of *luxO* in *V. cholerae* and other *Vibrio* species were incorrect, and thus previous descriptions of the phosphomimic *luxO* D47E should have been labeled *luxO* D61E (37). We also found this to be true for *V. campbellii* DS40M4 and thus refer to this allele as *luxO* D61E throughout this manuscript. The *luxO* D61E strain showed high levels of P*_vopN_-gfp* transcription, similar to the Δ*luxR* strain (Fig. 2C). Conversely, the Δ*luxO* strain showed diminished levels of P*_vopN_-gfp* transcription, similar to the wild-type strain at HCD (Fig. 2C). These patterns aligned with those of all previous studies of the QS regulatory epistasis (19–21).

We next used the reporter assay to test the role of the Exs regulatory cascade comprising ExsA, ExsD, ExsC, and ExsE proteins in the regulation of T3SS in *V. campbellii*. We designed transcriptional knockdowns of the *exs* genes using CRISPRi technology (41). In the *exsD* and *exsE* knockdown strains, GFP fluorescence from the *vopN* promoter was significantly increased compared to wild-type (Fig. 2D). This result was expected because, if similar to the *V. parahaemolyticus* model, a decrease in ExsD protein would free up ExsA protein to enable transcription activation, and a decrease in ExsE would free ExsC to interact with ExsD and thus release ExsA. In the *exsC* knockdown strain, GFP fluorescence was similar to that of wild-type (Fig. 2D). This result was expected because if the epistasis determined for *V. parahaemolyticus* is conserved in *V. campbellii*, a decrease in ExsC would result in more unbound ExsD to bind with ExsA and block transcription activation. We conclude that the effects of ExsC, ExsD, and ExsE knockdowns in *V. campbellii* follow a pattern that is predicted by the *V. parahaemolyticus* system.

### LuxR regulates the exsC *promoter*

**Fig 3:**
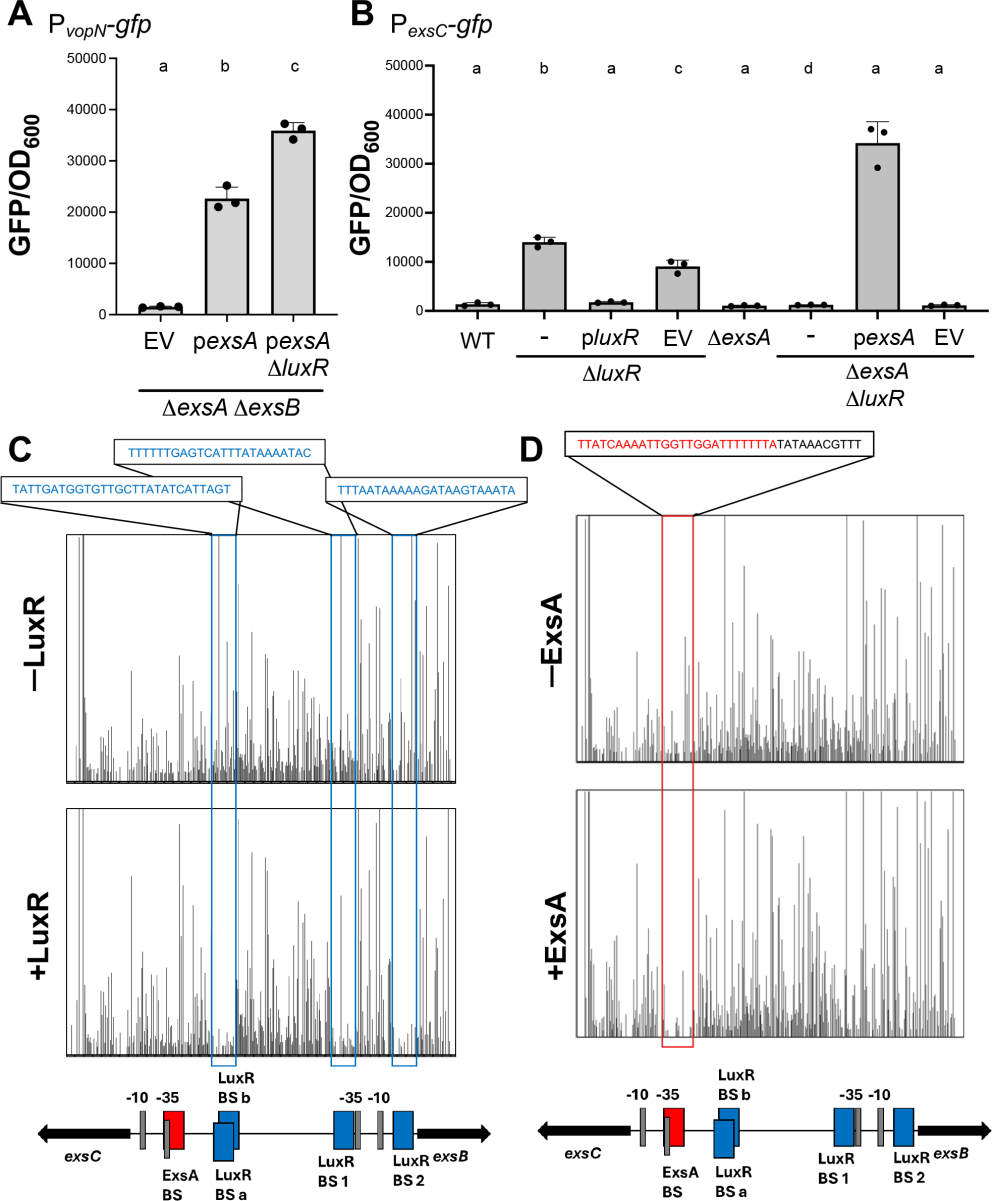
LuxR and ExsA bind and regulate *exsC.* (**A**) GFP reporter assay results are shown for strains Δ*exsA*Δ*exsB* and Δ*exsA*Δ*exsB* Δ*luxR* containing P*_vopN-gfp_* reporter (pPP26) and either the P*_tactheo_-exsA* expression plasmid (p*exsA*, pPP25) induced with 10 µM IPTG and 1 mM theophylline or an empty vector control plasmid (EV). (**B**) GFP reporter assay results are shown for strain Δ*exsA*Δ*exsB* containing the P*_exsC-gfp_* reporter (pPP51) and either no additional plasmid (–), P*_tactheo_-exsA* expression plasmid (p*exsA*, pPP25) induced with 10 µM IPTG and 1 mM theophylline, the P*_tactheo_-luxR* expression plasmid (p*luxR*, pPP60) induced with 1 mM IPTG and 1 mM theophylline, or an empty vector control plasmid (EV). For both panels, the error bars represent the standard deviation of the mean. A one-way ANOVA test was performed on normally distributed data (Shapiro–Wilk test), followed by Tukey’s multiple comparisons test. Different letters indicate significant differences between strains in pairwise comparisons (*P* < 0.05; *n* = 3). (**C, D**) DNaseI footprinting assays were performed with a 425 bp DNA probe corresponding to the sequence spanning the *exsC* and *exsB* promoters. The final concentration of the DNA probe used in the reaction mix was 20 nM. (**C**) A DNaseI-only treated control reaction (top; -LuxR) was compared with a reaction containing 0.25 µM LuxR (bottom, +LuxR). (**D**) A DNaseI-only treated control reaction (top, -ExsA) was compared with a reaction containing 1 µM ExsA (bottom, +ExsA). The red boxes indicate the ExsA-binding site (BS), and the blue boxes indicate LuxR BSs a, b, 1, and 2. The gray boxes indicate the −10 and −35 sites.

In *V. campbellii* BB120, the ~40 genes comprising the T3SS are on a pathogenicity island and are subdivided into four structural operons called T3SS I, II, III, and IV with promoters in front of the *vcrG*, *vopN*, *vscN*, and *exsD* genes, respectively (Fig. S3A) (19–21). ExsA is required for activation of T3SS gene expression of all four structural operons (21). Given the role of LuxR as a negative regulator of T3SS activation, we wanted to test if LuxR regulates the T3SS exclusively through repression of *exsA* transcription from the *exsBA* operon or if there are additional T3SS loci regulated by LuxR. We hypothesized that LuxR represses transcription from the four T3SS structural operons in addition to shutting down *exsA* transcription from the *exsBA* operon at HCD. To test our hypothesis, we designed an assay in which *exsA* transcription and translation were uncoupled from its native promoter, and T3SS gene expression was measured: i) the strain background is Δ*exsA* Δ*exsB*, and *exsA* was expressed ectopically from a plasmid, wherein transcription was under the control of an autonomous IPTG-inducible promoter (P*_tac_-exsA*), ii) translation of ExsA was also regulated through the placement of a theophylline-dependent riboswitch next to the promoter to regulate ribosome binding and translation (P*_tactheo_-exsA*) (42, 43), and iii) the P*_vopN_-gfp* reporter described above was used to monitor T3SS gene expression. Using this assay, ExsA expression was induced with 10 µM IPTG and 1 mM theophylline, which resulted in increased GFP production in the P*_tactheo_-exsA* strain compared to the vector control, as expected (Fig. 3A). The observed differences in *vopN-gfp* expression were not due to differences in growth rates (Fig. S2). To test our hypothesis that LuxR regulates T3SS via another mechanism beyond regulation of *exsA*, we compared the Δ*exsA* Δ*exsB* strain to the Δ*exsA* Δ*exsB*Δ*luxR* strain, both in the presence of induced ExsA expression. We observed higher transcription from the *vopN* promoter in the absence of LuxR compared to the presence of LuxR when ExsA was expressed via an autonomous promoter (Fig. 3A).

To test our hypothesis that LuxR binds to the T3SS structural promoters I–IV to regulate transcription, we performed bioinformatic analysis of LuxR binding sites on the BB120 genome (43). However, no binding sites were predicted at the I, II, III, or IV promoters. On the other hand, our bioinformatic analysis identified multiple LuxR sites at the *exsB* and *exsC* promoters (Fig. S3B). To further test these results, we performed *in vitro* DNaseI footprinting assay on the intergenic region between *exsC* and *exsB* with LuxR. We also performed differential RNA sequencing (dRNA-seq) to determine the precise position of transcription start sites (TSSs) of the T3SS promoters in *V. campbellii* (Fig. S3A). We observed DNaseI protection at the two LuxR predicted sites close to the *exsB* gene and at the two overlapping sites close to the *exsC* gene, as predicted in our bioinformatic analysis (Fig. 3C). We next constructed an *exsC* promoter fusion with a promoterless *gfp* cassette (integrated into the *V. campbellii* genome via Tn7 transposition) and assayed expression in the presence and absence of *luxR*. We observed that transcription from the *exsC* promoter was repressed by LuxR (Fig. 3B); complementation of *luxR* restored expression to levels similar to the parent strain background. The pattern of regulation of the *exsC* gene was further corroborated by RT-qPCR (Fig. S3D). From these data, we conclude that LuxR represses *exsC*.

### ExsA activates *exsC* but not *exsBA*

The ExsA-binding site is highly conserved across gram-negative species including *Pseudomonas aeruginosa, Yersinia pestis*, *Yersinia enterocolitica*, *Photorhabdus luminescens*, and *Aeromonas hydrophila* (44, 45). Using the ExsA consensus binding motifs determined in earlier studies by DNaseI footprinting assays in *P. aeruginosa,* we performed an *in silico* search for ExsA-binding sites across the T3SS pathogenicity island (Fig. S4A and B) (44, 45). We identified predicted ExsA-binding sites at the promoters of the four T3SS structural operons starting with *exsD*, *vopN*, *vscN,* and *vcrG* genes (Fig. S4B). Additionally, we found a predicted ExsA-binding site at the *exsC* promoter; however, no predicted ExsA-binding site was observed at the *exsB* promoter (Fig. S4B). To test our *in silico* results, we performed a DNaseI footprinting assay using the same *exsC–exsB* intergenic region, as tested with LuxR. The assay results showed that ExsA indeed only binds at the *exsC* promoter at the consensus binding site spanning the −35 site and an A-rich region upstream of it (Fig. 3D). No DNaseI protection by ExsA was observed near the *exsB* promoter. In addition, we assayed the role of ExsA in the activation of transcription from the *exsB* promoter via a P*_exsB_-gfp* fusion reporter (ectopic reporter on a plasmid backbone) in Δ*luxR* and Δ*exsA* Δ*luxR* strains. As a control, deletion of *luxR* led to significantly increased GFP expression compared to the wild-type strain, as shown previously (Fig. S4C). However, no significant difference was observed comparing the Δ*exsA* Δ*luxR* strain to the Δ*luxR* strain (Fig. S4C), indicating that ExsA does not impact transcription from the *exsB* promoter.

We proceeded to test the impact of ExsA binding to its site in the *exsC* promoter on *exsC* transcription. We used the P*_exsC_-gfp* reporter in the chromosome of a Δ*luxR* strain (to derepress the promoter) and assessed the effect of deletion of *exsA*. We observed that the Δ*exsA* Δ*luxR* strain had a significantly lower GFP expression compared to Δ*luxR* (Fig. 3B). Complementation of *exsA* under an autonomous promoter restored GFP expression to a level higher than that of the Δ*luxR* parent strain (Fig. 3B). Deletion of the ExsA-binding site in the Δ*luxR* strain significantly reduced transcription of P*_exsC_-gfp* to background levels (Fig. S4D). From these data, we conclude that ExsA binding to its predicted site at the *exsC* promoter activates transcription of *exsC*. We also conclude that ExsA does not bind nor activate the *exsB* promoter.

### LuxR post-translationally represses ExsA activity through ExsC

**Fig 4:**
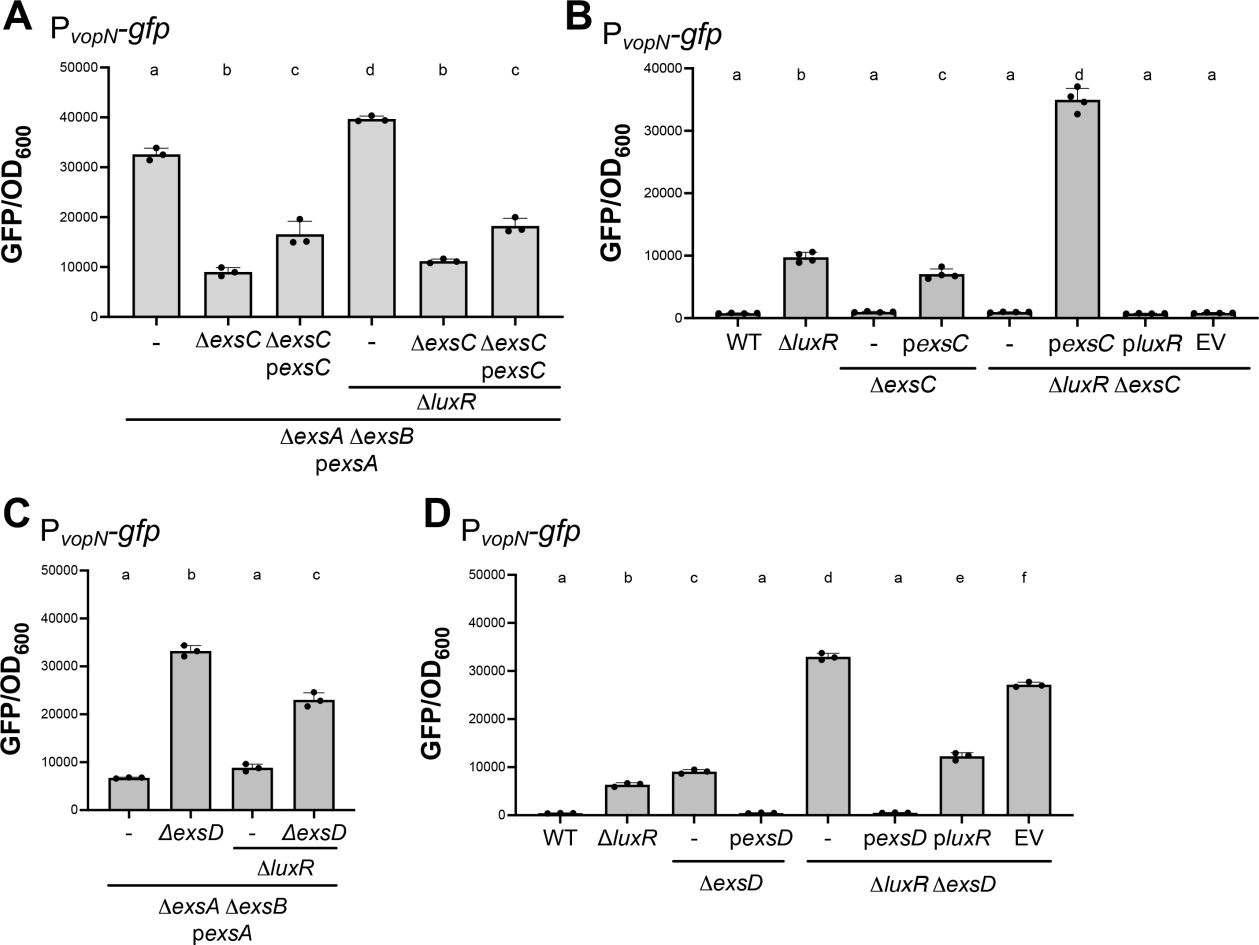
Deletion of *exsC* and *exsD* is epistatic to *luxR.* (**A, B**) GFP reporter assay results are shown for isogenic *V. campbellii* strains all with a Δ*exsA*Δ*exsB* background and containing either the P*_tactheo_-exsA* expression plasmid (p*exsA*, pPP25), or the P*_tactheo_-exsA* P*_tactheo_-exsC* double-expression plasmid (p*exsA,* p*exsC*, and pPP62), and the P*_vopN-gfp_* chromosomal reporter (pPP26). Other isogenic strain mutations include Δ*exsC*, Δ*luxR*, and complementation with the P*_tactheo_-exsC* expression plasmid (p*exsC*, pPP59), P*_tactheo_-luxR* expression plasmid (p*luxR*, pPP60), or the empty vector control plasmid (EV). ExsA alone/ExsA-ExsC were induced with 10 µM IPTG and 1 mM theophylline, ExsC alone was induced with 100 µM IPTG and 1 mM theophylline, and LuxR was induced with 1 mM IPTG and 1 mM theophylline. (**A, B**) Error bars represent the standard deviation of the mean. A one-way ANOVA test was performed on normally distributed data (Shapiro–Wilk test), followed by Tukey’s multiple comparisons test. Different letters indicate significant differences between strains in pairwise comparisons (*P* < 0.05; *n* = 3 (**A**) or *n* = 4 (**B**)). (**C, D**) GFP reporter assay results are shown for isogenic *V. campbellii* strains all with a Δ*exsA*Δ*exsB* background and containing the P*_tactheo_-exsA* expression plasmid (p*exsA*, pPP25) and the P*_vopN-gfp_* chromosomal reporter. Other isogenic strain mutations include Δ*exsD*, Δ*luxR*, and complementation with either the P*_tactheo_-exsD* expression plasmid (p*exsD*, pPP80), P*_tactheo_-luxR* expression plasmid (p*luxR*, pPP60), or the empty vector control plasmid (EV). ExsA was induced with 10 µM IPTG and 1 mM theophylline, ExsD was induced with 100 µM IPTG and 1 mM theophylline, and LuxR was induced with 1 mM IPTG and 1 mM theophylline. Error bars represent the standard deviation of the mean. A one-way ANOVA test was performed on normally distributed data (Shapiro–Wilk test), followed by Tukey’s multiple comparisons test. Different letters indicate significant differences between strains in pairwise comparisons (*P* < 0.05; *n* = 3).

In *P. aeruginosa* and *V. parahaemolyticus*, ExsC is a chaperone protein that binds the secretory protein ExsE during noninducing conditions (Fig. 1A). During inducing conditions, ExsE is secreted through the T3SS machinery, which drives the formation of the ExsC–ExsD complex (26, 28). This event releases ExsA, which binds T3SS gene promoters and activates their transcription (26, 28). The activation of *exsC* expression by ExsA is a regulatory mechanism by which ExsA keeps its anti-activator ExsD bound and sequestered away from itself (26, 28). Thus, we hypothesized that LuxR repression of *exsC* would promote the ExsA–ExsD interaction, decrease free ExsA protein, and thus decrease transcription of the T3SS structural genes. To test our hypothesis, we first tested the effect of *exsC* deletion on P*_vopN_-gfp* expression. In this experiment, we sought to separate LuxR regulation of *exsA* transcription and again utilized a background strain in which both *exsA* and *exsB* were deleted and *exsA* was induced from the autonomous P*_tactheo_* promoter (Δ*exsA* Δ*exsB* p*exsA*) using 10 µM IPTG and 1 mM theophylline. We observed that transcription from the *vopN* promoter was decreased in the absence of ExsC (Fig. 4A), which aligned with the predicted role of ExsC as an anti-anti-activator of ExsA. There was no significant difference comparing GFP expression in the presence or absence of LuxR in the Δ*exsA* Δ*exsB* Δ*exsC* P*_tactheo_-exsA* strain (Fig. 4A), indicating that the effects of LuxR regulation on *vopN* promoter expression were abrogated in the Δ*exsA* Δ*exsB* Δ*exsC* strain background. Induction of ectopic expression of both ExsA and ExsC using 10 µM IPTG and 1 mM theophylline was able to partially complement the activation of transcription from the *vopN* promoter, and we observed no significant difference in *vopN* transcription in the presence and absence of LuxR in the complementation strains (Fig. 4A). Thus, uncoupling LuxR regulation of *exsC* through ectopic expression of both the *exsA* and *exsC* genes completely abrogated LuxR regulation of T3SS.

We believe the partial complementation attained for *exsA* and *exsC* double expression via P*_tactheo_* was due to low expression of *exsC* at the induction used*,* even though *exsA* was optimally expressed at the same induction (Fig. 4A). To test our hypothesis, we also deleted and complemented *exsC* in a strain with *exsA* intact. We induced *exsC* using higher levels of inducers (100 µM IPTG and 1 mM theophylline). First, we observed that deletion and autonomous expression of *exsC* in a wild-type background increased GFP expression to a level similar to that of Δ*luxR* (Fig. 4B). Further, deletion of *exsC* in the Δ*luxR* background had low GFP levels, suggesting that even though *exsA* was derepressed, the absence of ExsC resulted in low *vopN* transcription. We hypothesize that this is due to excess ExsD protein (free of ExsC) binding up all of the ExsA protein. Also, in a Δ*luxR* background, deletion and autonomous expression of *exsC* produced levels of GFP that were maximal (Fig. 4B). These two results suggest that the highest levels of *vopN* occur in the absence of LuxR and autonomous expression of ExsC. We also assessed the role of ExsD, the putative anti-activator of ExsA. Deletion of *exsD* in the Δ*exsA* Δ*exsB* p*exsA* strain significantly increased *vopN* transcription that was independent of *luxR* (Fig. 4C). Complementation of *exsD* (induced using 100 µM IPTG and 1 mM theophylline) restored expression to wild-type levels (Fig. 4D). Of note, the expression of *luxR* in a Δ*luxR* Δ*exsD* background decreased *vopN* transcription to a level similar to that of Δ*exsD* (although significantly different). From these collective results and those above, we conclude that i) LuxR represses the transcription of *exsA* and *exsC*, ii) ExsC activates T3SS gene expression post-translationally, and iii) ExsD represses T3SS gene expression post-translationally. Also, our data show that there are three ways that expression of T3SS structural genes (like *vopN*) is maximized: i) when *exsA* is autonomously overexpressed, ii) when *exsD* is deleted and *luxR* is deleted, or iii) when *exsC* is overexpressed and *luxR* is deleted. Thus, there are two levels of ExsA regulation by LuxR: transcriptional and post-translational.

### LuxR and ExsA non-competitively bind to the *exsC* promoter

**Fig 5:**
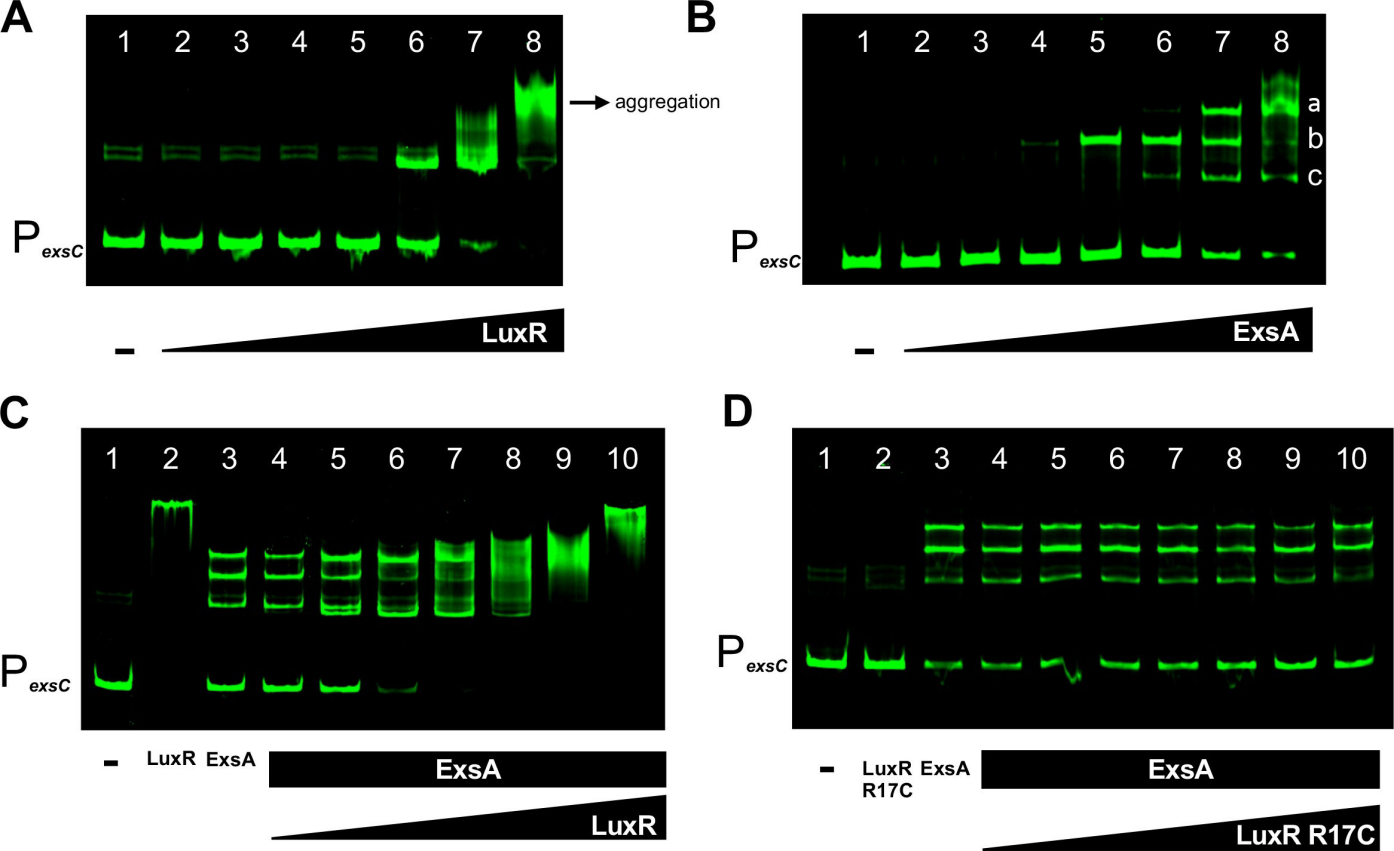
LuxR and ExsA bind the *exsC* promoter *in vitro*. (**A–D**) Electrophoretic mobility shift assays (EMSAs) were performed with a 200 bp DNA probe corresponding to the *exsC* promoter. The final concentration of DNA used was 2 nM. Lane 1, DNA probe only control. (**A**) Lanes 2–8, fivefold dilution series of LuxR from 500 nM to 0.032 nM. (**B**) Lanes 2–8, fourfold dilution series of ExsA from 2 µM to 0.488 nM. Letters a, b, and c indicate different shifted band patterns. (**C, D**) Lane 2, LuxR or LuxR R17C alone at 700 nM and 1 µM concentrations, respectively. Lane 3, ExsA alone at 500 nM concentration. Lanes 4–10, ExsA was first added at a concentration of 500 nM, followed by addition of either LuxR in a twofold dilution series from 700 nM to 10.94 nM, or LuxR R17C in a twofold dilution series from 1 µM to 15.625 nM. The original images for each gel have been included in the Fig. S8A through D.

We sought to investigate the molecular mechanism by which LuxR represses the *exsC* promoter. We hypothesized that LuxR blocks or alters ExsA binding to the *exsC* promoter. To assay this, we first tested binding patterns of LuxR and ExsA using *in vitro* electrophoretic mobility assays (EMSAs) with a probe corresponding to the *exsC* promoter (Fig. S3B). LuxR bound the *exsC* promoter substrate with shifts observed starting at 20 nM LuxR (Fig. 5A). We also observed possible LuxR aggregation at a high concentration of 500 nM (Fig. 5A); this is commonly observed in other published studies of LuxR at >500 nM concentrations on any substrate, including nonspecific DNA sequences (46–48). ExsA bound the *exsC* promoter with a higher affinity, with a shift in DNA migration observed starting at 7.81 nM protein concentration (Fig. 5B). The binding of ExsA at the *exsC* promoter shows multiple shifts observed at higher concentrations of the protein (Fig. 5B), possibly indicating DNA binding activity by ExsA. DNA binding by AraC family proteins as a dimer, and subsequent binding of the DNA has been shown in previous studies of ExsA homologs in *P. aeruginosa* and *Y. pestis* (44). The DNA-binding pattern of ExsA on the *exsC* promoter substrate suggests that ExsA binds the *exsC* promoter strongly. As negative controls, we performed EMSAs with a probe corresponding to the *mutS* locus to test for LuxR and ExsA specificity. We observed no shift for either ExsA or LuxR at the control locus even at high concentrations of 3 µM and 500 nM, respectively (Fig. S5A and B). To further test for specificity of ExsA binding at the *exsC* promoter, we performed competitive EMSAs wherein the fluorescently labeled *exsC* probe was competed with increasing concentrations of either unlabeled *mutS* probe or unlabeled *exsC* probe. We observed that ExsA binding at the labeled *exsC* probe (2 nM concentration) was not disrupted by the unlabeled *mutS* probe even at a high concentration of 100 nM (Fig. S5C). On the other hand, competing the labeled *exsC* probe (2 nM concentration) with an increasing concentration of unlabeled *exsC* probe disrupted ExsA interaction at 20 nM and 100 nM concentrations (Fig. S5D). These results together show that binding of both ExsA and LuxR at the *exsC* promoter is highly specific.

**Fig 6:**
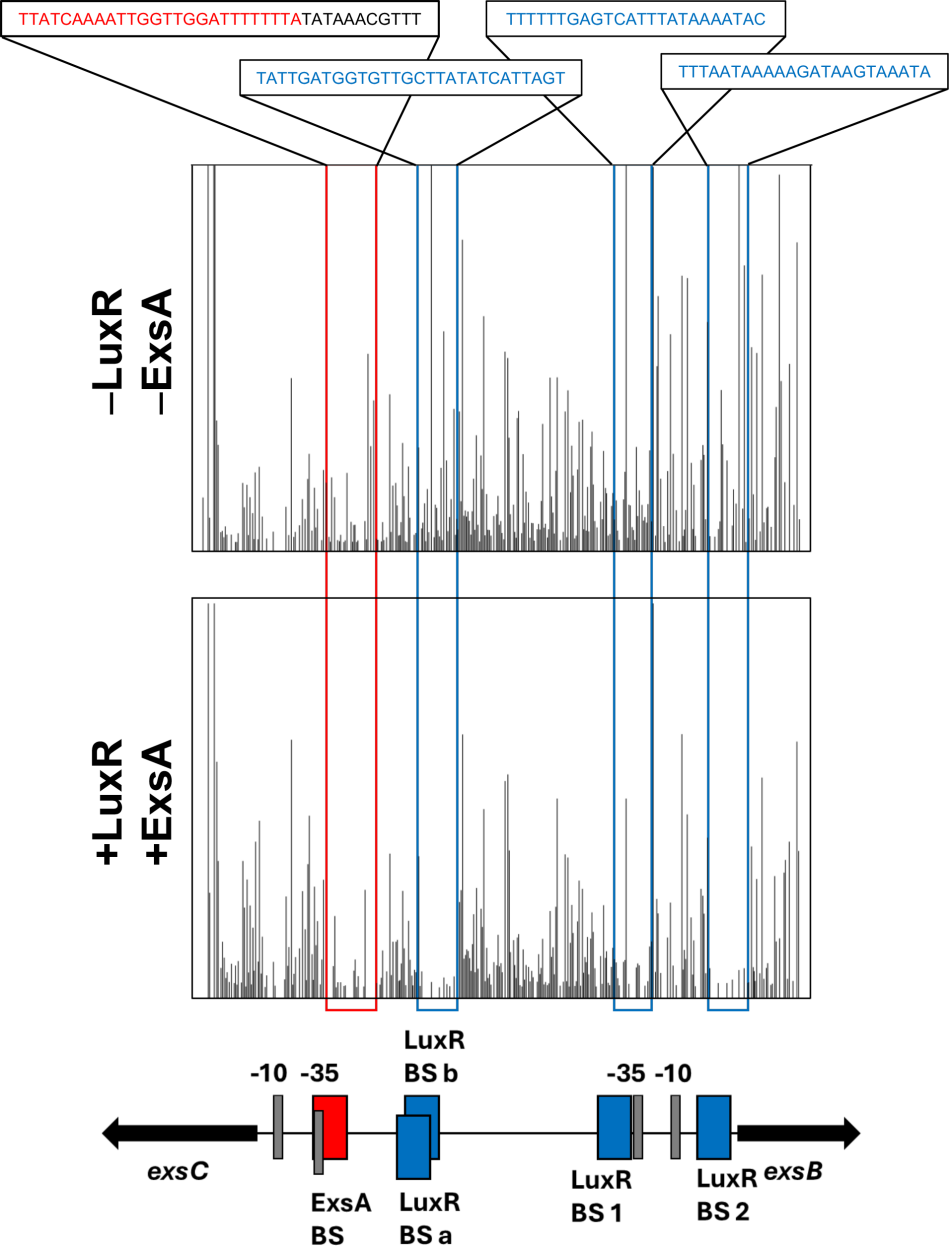
LuxR and ExsA co-bind at the *exsC* promoter. DNaseI footprinting assays were performed with a 425 bp DNA probe corresponding to the sequence spanning the *exsC* and *exsB* promoters. The final concentration of the DNA probe used in reaction mix was 20 nM. DNase I-only treated probe (top) was compared with the probe treated with 1 µM ExsA and 0.25 µM LuxR, followed by DNaseI treatment (bottom). Protection by ExsA was observed along the predicted ExsA-binding site at the *exsC* promoter proximal to the −35 site. Protection by LuxR was observed along the predicted LuxR-binding site at the *exsC* and *exsB* promoters.

Because we observed that LuxR and ExsA both bound at the *exsC* promoter, we hypothesized that LuxR represses the *exsC* promoter by disrupting ExsA binding, which we previously observed to be required for *exsC* activation. To test our hypothesis, we performed competitive gel shift assays with LuxR and ExsA on the *exsC* promoter substate (Fig. 5C and D). We performed the competitive gel shift assays in two ways: i) by pre-incubating DNA probe with ExsA and then titrating in LuxR (Fig. 5C, S6B) or ii) by pre-incubating the DNA probe with LuxR and then titrating in ExsA (Fig. S6A). In both cases, we observed shift patterns that suggested co-binding of the probe by ExsA and LuxR instead of competition between the proteins (Fig. 5C; Fig. S6A and B). Conversely, there was no observable change in the ExsA shift pattern when we titrated in increasing concentrations of DNA-binding mutant protein LuxR R17C (Fig. 5D). These results suggest that the binding pattern observed is due to LuxR-specific DNA interactions at the *exsC* promoter and not due to LuxR interacting solely with ExsA. We next performed competitive DNase I footprinting assays wherein we incubated a DNA probe spanning the *exsB* and *exsC* promoters with ExsA, followed by addition of LuxR (Fig. 6). We observed that ExsA remained bound at the *exsC* promoter even after addition of LuxR (Fig. 6), once again suggesting that LuxR does not disrupt ExsA binding at the *exsC* promoter. These results disproved our hypothesis that LuxR binding alters ExsA binding at the *exsC* promoter.

### Transcription of *exsC* requires an element upstream of the ExsA binding site

**Fig 7:**
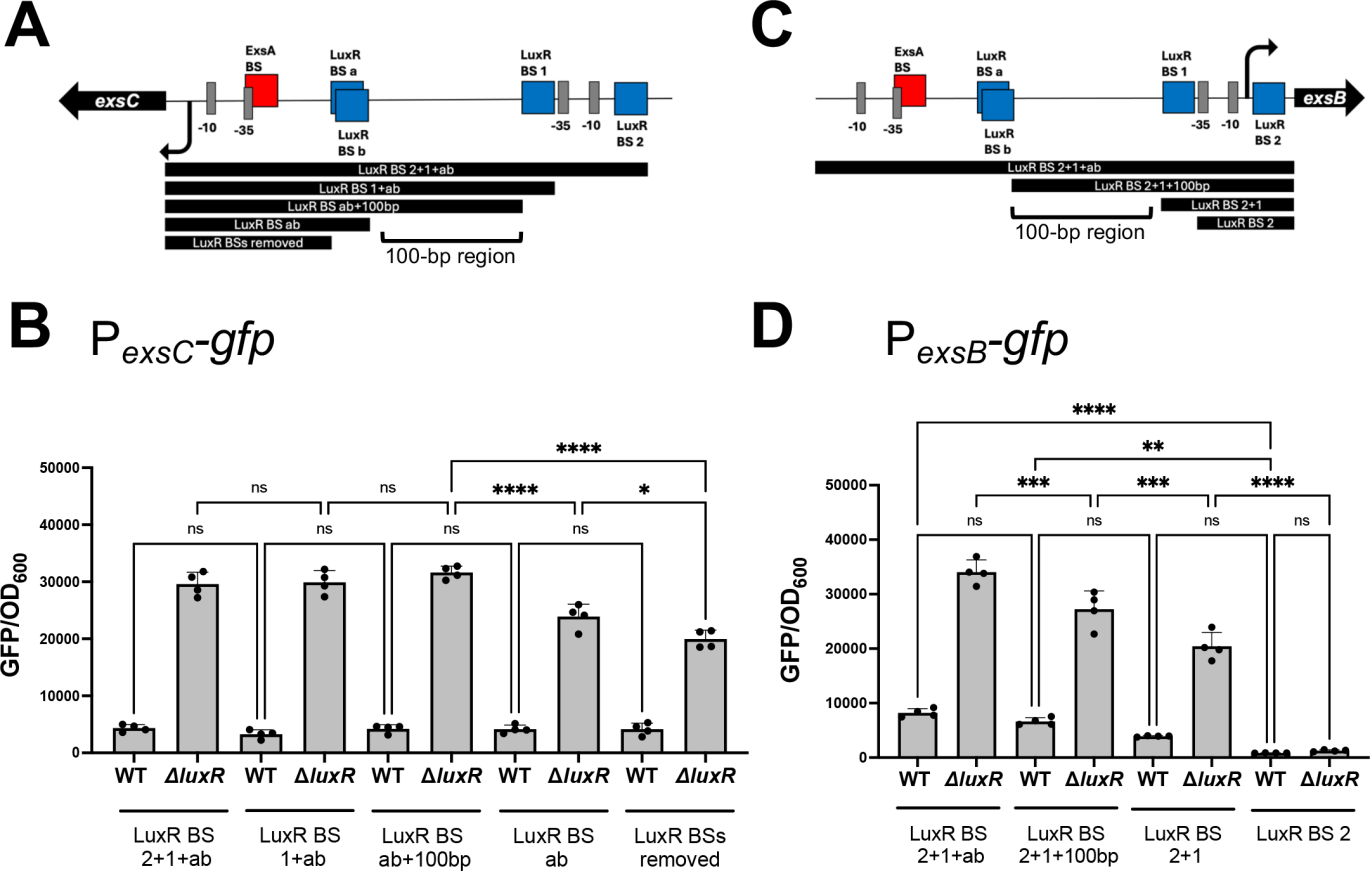
LuxR represses *exsC* and *exsB* transcription through the promoter-proximal LuxR-binding sites. (**A, C**) Schematic of different lengths of *exsC* or *exsB* promoters fused to the *gfp* cassette. (**B, D**) GFP reporter assays with the *gfp* cassette fused to various lengths of the *exsC* or *exsB* promoters shown in (**A**) and (**C**). The P*_exsC_-gfp* reporters (pPP51, pPP77, pPP83, pPP84, and pPP85) are fused to the chromosome, whereas the P*_exsB_-gfp* reporters (pPP07, pPP82, pPP86, and pPP87) are on ectopic plasmids. Error bars represent the standard deviation of the mean. A one-way ANOVA test was performed on normally distributed data (Shapiro–Wilk test), followed by Tukey’s multiple comparisons test. Different letters indicate significant differences between strains in pairwise comparisons (*P* < 0.05; *n* = 4).

Based on our above results, we next hypothesized that LuxR binding disrupted a different aspect of *exsC* transcription. This was supported because the LuxR “a/b” sites were 61 bp upstream from the ExsA-binding site. To determine the roles of the multiple LuxR-binding sites in the *exsC* promoter, we constructed *gfp* reporter fusions with different lengths of the promoters by sequentially deleting each LuxR-binding site and comparing expression in wild-type and Δ*luxR* strains (Fig. 7A). Different lengths of the *exsC* promoter tested showed that there was a decrease in *exsC* transcription when the intergenic region is missing. Specifically, there is a 100 bp region between the overlapping LuxR binding sites “a/b” and site “1.” When this region was removed from the promoter, a significant reduction in transcription of *exsC* was observed (Fig. 7B). Deletion of sites “a/b” alone at P*_exsC_* also showed a similar phenotype (Fig. S4D). Therefore, we hypothesize that LuxR represses *exsC* transcription by occluding site(s) important for its maximal activation. However, we can conclude that LuxR binding sites “1” and “2” did not have a role in *exsC* transcription (Fig. 7B).

We also performed a similar experiment testing different lengths of the *exsB* promoter using a *gfp* reporter (Fig. 7C). We observed that LuxR-binding sites “a/b” and the 100 bp region between sites “a/b” and site “1” play a role in maximal activation of *exsB* transcription (Fig. 7D). We also observed that deletion of site “1” completely abrogated *exsB* transcription (Fig. 7D). Deletion of LuxR-binding sites “1” and “2” individually at P*_exsB_* also showed similar phenotypes (Fig. S4C), indicating that these sites are essential for transcription. We hypothesize that this is due to their proximity to the −35 and −10 promoter elements (Fig. 7C). From these results, we conclude that maximal transcription of *exsC* and *exsB* requires the presence of the intergenic region between the genes and the absence of *luxR*. Further, our data collectively indicate that LuxR does not competitively bind with ExsA, but rather that the LuxR “a/b” sites overlap another DNA element required for *exsC* transcription activation.

## DISCUSSION

**Fig 8:**
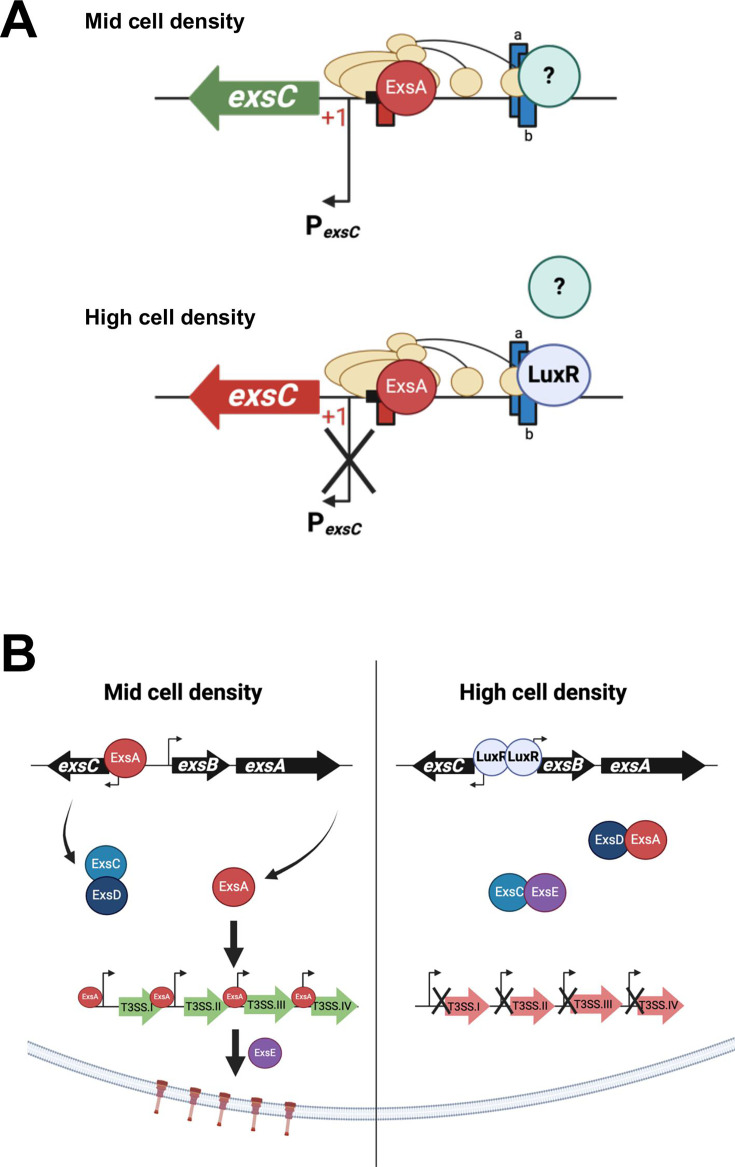
Models of the dual mechanism of T3SS regulation by LuxR. (**A**) Proposed model for activation of *exsC* transcription by class II activator ExsA and an unidentified class I activator (“?”) at mid-cell density. LuxR binds and displaces the unidentified class I activator by binding to LuxR sites “a/b” at high cell density. **B**) At mid-cell density, the absence of LuxR (and likely other regulators) enables transcription of the *exsC* promoter by ExsA, which releases existing ExsA bound by ExsD. ExsA activates transcription of the four T3SS structural operons, enabling synthesis of the T3SS needles. At high cell density, LuxR represses transcription of *exsA* from the *exsBA* promoter and represses transcription of *exsC*. Lower levels of ExsC enable ExsD to bind to ExsA and prevent its activity as a transcriptional activator of T3SS genes.

Our genetic epistasis and biochemical assays have uncovered a new regulatory role of the master QS transcriptional regulator LuxR in controlling T3SS expression: LuxR represses transcription of the gene encoding the anti-anti activator ExsC (Fig. 8B). Our model proposes a dual mechanism of regulation controlling the expression of the T3SS structural genes in response to changes in the cell density, at HCD: i) LuxR represses transcription of *exsBA*, and thus the master transcription factor ExsA is not produced; ii) LuxR represses transcription of *exsC,* and the decrease in ExsC protein promotes formation of the ExsD–ExsA complex and thus inactive ExsA protein (Fig. 8B). While LuxR-mediated repression of *exsA* is the major regulatory mechanism, inactivation of ExsA activity post-translationally does play a quantifiable role in shutting down T3SS at HCD. Our data suggest that this mechanism of regulation is a coherent type II feedforward loop wherein LuxR inhibits ExsA and ExsC separately, both of which act as activators of the T3SS structural genes (49).

Our promoter–reporter truncation experiments also strongly indicate the presence of at least one additional activator required for the maximal transcription of *exsC*. Based on the locations of the ExsA-binding site and the undefined upstream element, we hypothesize that the *exsC* promoter is activated through two activators at the promoter (illustrated in our model in Fig. 8A): i) ExsA, the promoter-proximal activator that functions as a class II activator as its binding site overlaps the −35 element and ii) an unknown promoter-distal class I activator that contacts the CTD of the α−subunit of RNA polymerase (Fig. 8A) (50). We hypothesize that LuxR repression of the *exsC* promoter occurs through the disruption of binding of the unidentified class I activator. Further, our results indicate a regulatory role for an unidentified activator at the *exsB* promoter (Fig. 8A) as well. There are numerous prior studies detailing additional regulators of *exsA* in *Vibrio* species. These include the following: i) H-NS, a histone-like nucleotide-binding protein and global regulator of gene expression that represses T3SS transcription by binding along the length of the promoter regions of T3SS1 genes *exsE*, *exsC*, *exsB*, and *exsD* (27, 47); ii) HlyU, a global activator of virulence genes that relieves repression of DNA transcription by removing H-NS, thereby acting as an activator of T3SS (51); iii) LuxT, a global low cell density (LCD) regulator that activates ExsA expression indirectly by repressing *swrZ* expression (52); and iv) SwrZ, a regulator of swarming motility repressing ExsA expression (52). It is unclear which of these activators and repressors may be playing roles in *exsC* expression, although this would be a target for future studies of this intergenic region.

There is apparent homology in the sequence and function of *exs* genes in *Vibrio* and *Pseudomonas*, yet the synteny and regulatory patterns differ in numerous ways (Fig. S6) (26, 27, 33, 44). In both *V. parahaemolyticus* and *V. campbellii*, the expressions of *exsB* and *exsA* are coupled and distinct from *exsCE*, whereas in *Pseudomonas*, *exsECB* are co-transcribed and distinct from *exsA* (19–21, 26, 33). In *V. parahaemolyticus*, ExsA activates its own expression from both the *exsB* and *exsA* promoters (33), yet in *V. campbellii*, ExsA does not activate its own expression based on the data we present in this study. This regulatory mechanism is more similar to that of *P. aeruginosa* in which transcription from P*_exsA_* is activated by global regulators Vfr, Fis, and VqsM, but not ExsA (26). There are also similarities in regulatory patterns among the three organisms. ExsA activates the expression of its own anti-activator ExsD, thereby regulating its own activity through a feedback loop (26, 33). ExsC and ExsE are also activated by ExsA in *P. aeruginosa*; thus, it would be relevant to test whether *V. parahaemolyticus* ExsA also activates *exsCE*.

During our examination of the divergent *exsBA* and *exsCE* promoters, we noted that the T3SS promoters differ from other σ70 promoters, in that the spacer sequence between the −10 and −35 sites is longer than the canonical 17 +/– 1 bp length and tends to be closer to 20–22 bp in length instead (Fig. S4A and B). The *vopN* promoter has the exact −10 and −35 consensus sites for σ70 promoters, TATAAT and TTGACA, respectively (Fig. S4B). The other T3SS promoters have approximately 50% homology to the consensus sites (Fig. S4B). The longer than canonical spacer region in the case of ExsA-regulated promoters is reminiscent of MerR promoters. The MerR promoters have a 19 bp spacer sequence between −10 and −35 sites (53). The binding of MerR to DNA under activating conditions causes a distortion in the DNA backbone so as to reorient the −35 and −10 sites, which allows them to interact positively with the RNA polymerase σ70 subunit (53). We hypothesize that ExsA binding is important to compensate for the weak −35 and −10 sites and the longer than usual spacer sequence. ExsA could be playing a similar role in which it brings the −35 site closer to the −10 site to match the consensus 17 bp spacer region and helps recruit RNA polymerase σ70 subunit to the ExsA-dependent promoters. The *exsB* promoter with no predicted ExsA-binding site differs from the other T3SS promoters, in that it has the canonical 17 +/– 1 bp spacer sequence between its −35 and −10 sites; also there is a conspicuous lack of the A-rich region upstream of the −35 site, as is present in the other T3SS promoters with predicted ExsA-binding sites.

The ExsA-binding site has two important features, an A-rich region and the −35 site downstream of it with a 13 bp gap in between (Fig. S4A and B). In *P. aeruginosa*, ExsA binds DNA as a monomer at a half site and recruits a second monomer to bind the other half site, thereby dimerizing post-DNA binding (44). Separate binding events by ExsA monomers followed by dimerization leads to multiple shifts observed in gel shift assays with *P. aeruginosa* T3SS promoters including P*_exsC_*, P*_exsD_*, and P*_exoT_* (44). Thus, the multiple shifts we observed of ExsA binding at P*_exsC_* could be indicative of dimerization or polymerization of ExsA post-DNA binding, leading to DNA twisting or bending for transcription activation (Fig. 5B). The ExsA consensus binding site has been observed at all four structural promoters of the T3SS, indicating the expected necessary role of ExsA in activating transcription from these promoters (Fig. S4B).

The results of our studies here and combined with those of previous studies show that the QS system in *V. campbellii* shuts down the T3SS at HCD via two separate mechanisms to block ExsA activity: transcriptionally and post-translationally (Fig. 8B). However, the evolutionary advantage for the repression of T3SS synthesis and activity at HCD is unclear. We speculate that the expression of T3SS at low- to mid-cell density is advantageous at some stage during host infection. There is precedence for this: *V. cholerae* temporally controls virulence during infection (54). At late-stage infection, the *V. cholerae* QS system shuts down virulence genes and turns on genes that favor cell dispersal either to form new foci of infection in the intestine or to exit the host and re-enter the aquatic environment (54). We postulate that the *V. campbellii* QS system shuts down virulence in late-stage infection for similar reasons: dispersal of cells to exit the host and re-enter the aquatic environment in search of new hosts to colonize or to continue the planktonic lifestyle. Experimental assessment of these hypotheses may lead to a better understanding of the *V. campbellii* shrimp infection mechanisms and adaptation to different niche conditions.

## MATERIALS AND METHODS

### Bacterial strains and media

The *E. coli* S17-1λ*pir* strain was used for cloning purposes, and the *E. coli* BL21 (DE3) strain was used for overexpression and purification of all proteins. *Escherichia coli* strains were cultured at 37°C with shaking (250–275 RPM) in lysogeny broth (LB) medium with 100 mg*/*mL kanamycin, 100 mg*/*mL gentamycin, and*/*or 10 mg*/*mL chloramphenicol when selection was required. *V. harveyi* BB120 was recently reclassified as *Vibrio campbellii* BB120 (also known as, ATCC BAA-1116) (9). BB120 and derivatives were cultured at 30°C with shaking (250–275 RPM) in Luria Marine (LM) medium with 100 mg*/*mL kanamycin, 10 mg*/*mL chloramphenicol, and*/*or 50 mg*/*mL polymyxin B when selection was required. Plasmids were transformed into electrocompetent *E. coli* S17-1λ*pir* cells and subsequently conjugated into *V. campbellii* strains. *V. campbellii* exconjugants were selected using polymyxin B (50 U*/*mL). In case of strains transformed with inducible plasmid pPP25 and pPP62, 10 µM IPTG, and theophylline at concentrations of 10 µM, 100 µM, and 100 µM was used to induce the cells.

### GFP reporter assays

Endpoint GFP reporter assays were performed. Bacterial cultures were inoculated in 3 mL LM and 15 mM MgSO4, and grown shaking at 30°C and 275 RPM overnight. About 1 mL of each culture was centrifuged for 3 minutes at 13,000 rpm to pellet down cells. The cell pellets were resuspended in 1 mL 1X saline PBS. The optical density was measured at 600 nm wavelength using either the Biotek Cytation3 plate reader or the Synergy H1 plate reader. The GFP signal was read at 485 nm excitation wavelength and 528 nm emission wavelength. Autogain function was performed for automatic adjustment of the GFP signal per strain per plate assay. The GFP signal obtained was normalized to growth by dividing GFP reads by OD_600_ reads.

### Growth curve assays

Bacterial cultures were inoculated in 3 mL LM and grown shaking at 30°C and 275 RPM overnight. Each culture was diluted 1:1,000 in 1 mL LM and 15 mM MgSO_4_. For strains requiring induction, an appropriate concentration of IPTG and theophylline was added. About 200 µL of each culture was pipetted into a clear 96-well plate and grown shaking at 30°C in the Synergy H1 plate reader for 18 hours of overnight growth. The optical density was recorded every 30 minutes measured at a wavelength of 600 nm.

### Molecular and chemical methods

PCR was performed using Phusion HF polymerase (New England Biolabs) and Iproof HF polymerase (BioRad). All oligonucleotides were ordered from Integrated DNA Technologies (IDT). PCR products and plasmids were sequenced using Eurofins Genomics. Cloning procedures are available upon request. DNA samples were resolved using 1% agarose (1 × TBE). Unless otherwise noted, data are plotted for triplicate independent experiments. Symbols on graphs represent the mean values, and error bars are standard deviations. Statistical analyses were performed with GraphPad Prism version 10.2.0. Additional information about statistical analyses is included in the figure legends. CRISPRi knockdown constructs were generated as described (41) with targeting small guide RNAs against *exsC*, *exsD*, and *exsE*.

### Construction of deletion/epitope-tagged strains

All *V. campbellii* BB120 and KM669 derivative strains in this study were constructed following a previously published technique (46). Briefly, the pRE112 suicide vector was used to construct unmarked deletions or insertions of epitopes in which 1,000 bp of the upstream and downstream flanking sequence was cloned into pRE112. The pRE112 derivatives were conjugated into *V. campbellii* and selected on chloramphenicol to induce chromosomal recombination of the plasmid. Subsequently, the plasmid was excised via counterselection on 15% sucrose. Cells in which the plasmid excision yielded a non-WT locus were detected via colony PCR. All gene deletions were confirmed by DNA sequencing through Eurofins.

### Expression and purification of LuxR protein

LuxR, His-LuxR, and FLAG-LuxR were purified as previously described (43, 46–48, 55) in BL21(DE3) cells containing either pJV079 (wild-type *luxR*) or pJV206 (*luxR* R17C).

### Expression and purification of ExsA protein

ExsA was purified by overexpressing N terminal 6X His-tagged ExsA (pPP43 vector ordered from BioTwist) in *E. coli* BL21(DE3) cells. The purification protocol is a modified version of a previously described protocol (29). The cells were initially grown at 37°C to an OD_600_ = 0.6–0.8, and ExsA overexpression was induced by using 1 mM IPTG for 4 hours. Induced cell pellets were resuspended in lysis buffer (20 mM Tris–HCl pH 8.0, 500 mM NaCl, 20 mM imidazole, 1 × protease inhibitors, 1 mM PMSF, 0.2 mg/mL DNaseI (GoldBio), 1 × FastBreak (Millipore)) and incubated at room temperature for 35 minutes. Tween 20 was added to a final concentration of 0.5% following lysis. The protease inhibitor mix included the following: 0.07 mg/mL phosphoramidon (Santa Cruz), 1.67 mg/mL AEBSF (DOT Scientific), 0.07 mg/mL pepstatin A (DOT Scientific), 0.07 mg/mL E-64 (Gold Bio), and 0.06 mg/mL bestatin (MPbiomedicals/Fisher). The clarified lysate was loaded onto a 5 mL His-Trap Ni-NTA column (GE Healthcare Life Sciences) using an Akta Pure (GE Healthcare Life Sciences). The protein was eluted from the column using a linear gradient of elution buffer (25 mM Tris–HCl pH 8.0, 500 mM NaCl, 500 mM imidazole). Fractions were analyzed by SDS-PAGE to confirm the presence of 6xHis-ExsA and pooled together. Pooled fractions were concentrated using 10 kDa cutoff centrifugal filters (Sartorius) and dialyzed overnight in storage buffer (20 mM Tris–HCl pH 8.0, 500 mM NaCl, 1 mM DTT, 0.5% Tween 20). Dialyzed protein was aliquoted, snap-frozen in liquid N2, and stored at −80◦C.

### Electrophoretic mobility shift assays

Primers to amplify over 200 bp of the promoter regions of exsB and exsC were ordered from IDT, where the 5’ end of one primer among the pair was labeled with IRD800CWN. PCR amplification was used to produce promoter substrates. The labeled and amplified DNA probe was incubated for 30 minutes in a 15 mL reaction mixture containing either 20X stock ExsA binding buffer (20 mM Tris [pH 7.5], 100 mM KCl, 2 mM dithiothreitol [DTT], 2 mM EDTA, 5% glycerol), or 10X stock LuxR binding buffer (10 mM HEPES [pH 7.5], 100 mM KCl, 2 mM dithiothreitol (DTT), 200 mM EDTA), 10 ng/mL poly(dI-dC), 0.1 mg/mL bovine serum albumin (BSA), and the desired protein diluted in either ExsA dilution buffer (1X stock ExsA binding buffer), or LuxR dilution buffer (20 mM imidazole [pH 7.5], 3000 mM NaCl, 0.5 mM EDTA, 1 mM DTT, and 5% glycerol). The reaction mixtures were separated on 6% TGE (25 mM Tris, 0.25 M glycine, 1 mM EDTA)-polyacrylamide native gels. Binding reactions were performed using either 20 nM labeled DNA or 20 nM unlabeled, bringing the final concentration of the DNA probe to 2 nM in the reaction. Poly(dI-dC) (10 ng/mL, Sigma) was used as nonspecific competitor DNA in all cases. For competitive binding experiments, reactions were supplemented with the secondary protein in binding buffer and incubated at room temperature for an additional 30 minutes.

### qRT-PCR, RNA extraction, and differential RNA-seq

Strains were inoculated in 5 mL LM and grown overnight shaking at 30°C at 275 RPM. Each strain was back-diluted 1:1,000 in LM and 15 mM MgSO_4_ and grown shaking at 30°C at 275 RPM until they reached an OD_600_ =  1.0. Two milliliter cells were collected by centrifugation at 3,700 RPM at 4°C for 10 minutes, the supernatant was removed, and the cell pellets were flash-frozen in liquid N_2_ and stored at −80°C. RNA was isolated from pellets using a TRIzol/chloroform extraction protocol and treated with DNase via the DNA-free DNA Removal Kit (Invitrogen), as previously described. Quantitative reverse transcriptase real-time PCR (RT-qPCR) was used to quantify transcript levels of T3SS genes in different regulatory conditions and was performed using the SensiFast SYBR Hi-ROX One-Step Kit (Bioline) according to the manufacturer’s guidelines. Primers were designed to have the following parameters: amplicon size of 100 bp, primer size of 20–28 bases, and melting temperature of 55°C–60°C. All reactions were performed using a Step-One Real PCR system with 0.4 µM of each primer and 200 ng of template RNA (20 µL total volume). All RT-qPCR experiments were normalized to the internal standard *hfq* gene. The ΔΔ*C_T_* or standard curve methods were used to analyze data from at least three independent biological replicates with two technical replicates each.

For dRNA-seq, cells were collected as described above but without MgSO_4_. The cDNA libraries were constructed as described previously (54) by Vertis Biotechnology AG (Freising, Germany) and sequenced using an Illumina NextSeq 500 machine in the single-read mode (75 bp read length). Reads were mapped and transcription start sites determined, as described previously (56). The raw, demultiplexed reads and coverage files have been deposited in the National Center for Biotechnology Information Gene Expression Omnibus with accession code GSE182898.

### DNase I footprinting assay

The promoter region for transcription activation of the *exsC* and *exsB* genes with LuxR- and ExsA-binding sites in *V. campbellii* BB120 (425 bp) PCR-amplified using a forward primer with a 5′-FAM fluorescent tag (IDT) and a reverse primer with a 5′-HEX fluorescent tag (IDT). DNase I (NEB) concentration was optimized by assessing the activity of DNase I across a range of concentrations (1 x-1024x, diluted with water), and the optimal concentration for the DNase I enzyme and buffer conditions was determined to be 128-fold dilution. Footprinting reactions were set up in the following conditions: 50 mM Tris-HCl pH 7.5, 10 mM MgCl_2_, 50 mM KCl, 0.1 mg/mL BSA, 1 mM DTT, 5% glycerol, 20 nM DNA probe, and either no protein or 1 µM ExsA or 0.25 µM LuxR or both. Reactions were incubated at room temperature for 15 minutes. Subsequently, 5 µL of DNase I (diluted 128-fold) was added to each reaction and incubated for 15 minutes at room temperature. To stop the digestion reaction, 25 µL 0.5 M EDTA pH 8.0 was added. DNA fragments were recovered using Qiagen MinElute PCR clean-up columns and eluted into black Eppendorf tubes. DNA fragments were analyzed with Genewiz Azenta Life Sciences using their Fragment analysis service and LIZ 500 DNA standard ladder. Peak Scanner software v1.0 was used for data analysis.

### Generation of the ExsA consensus binding site

Multiple sequence alignment data for the ExsA-binding site sequences were extracted from the previously published ExsA-dependent promoter sequences and putative T3SS promoter sequences from *Pseudomonas aeruginosa*, *Photorhabdus luminescens,* and *Aeromonas hydrophila* (44) using which a position weight matrix (PWM) was constructed. The PWM was searched against the *Vibrio campbellii* strain BB120 genome sequence using the FIMO (FInd MOtif) tool (57) from MEME suite version 5.1.0. Using the search hits with qval ≤0.01, the process of PWM construction followed by FIMO search was repeated iteratively until no more additional hits could be found. The sequence conservation among the significant matches to the ExsA motif was visualized as a sequence logo generated using Seqlogo version 2.8 (58).

## SUPPLEMENTAL MATERIAL

Supplemental Material mbio.00106-25-s0001.pdf Figures S1-S8.

ASM does not own the copyrights to Supplemental Material that may be linked to, or accessed through, an article. The authors have granted ASM a non-exclusive, world-wide license to publish the Supplemental Material files. Please contact the corresponding author directly for reuse.
