# Supplementary material for: Quorum sensing employs a dual regulatory mechanism to repress T3SS gene expression
Source: mBio. 2025 Feb 25;16(4):e00106-25. doi: 10.1128/mbio.00106-25 (PMC11980564; doi:10.1128/mbio.00106-25)
Supplement: Supplemental Material — Figures S1-S8. [file mbio.00106-25-s0001.pdf]

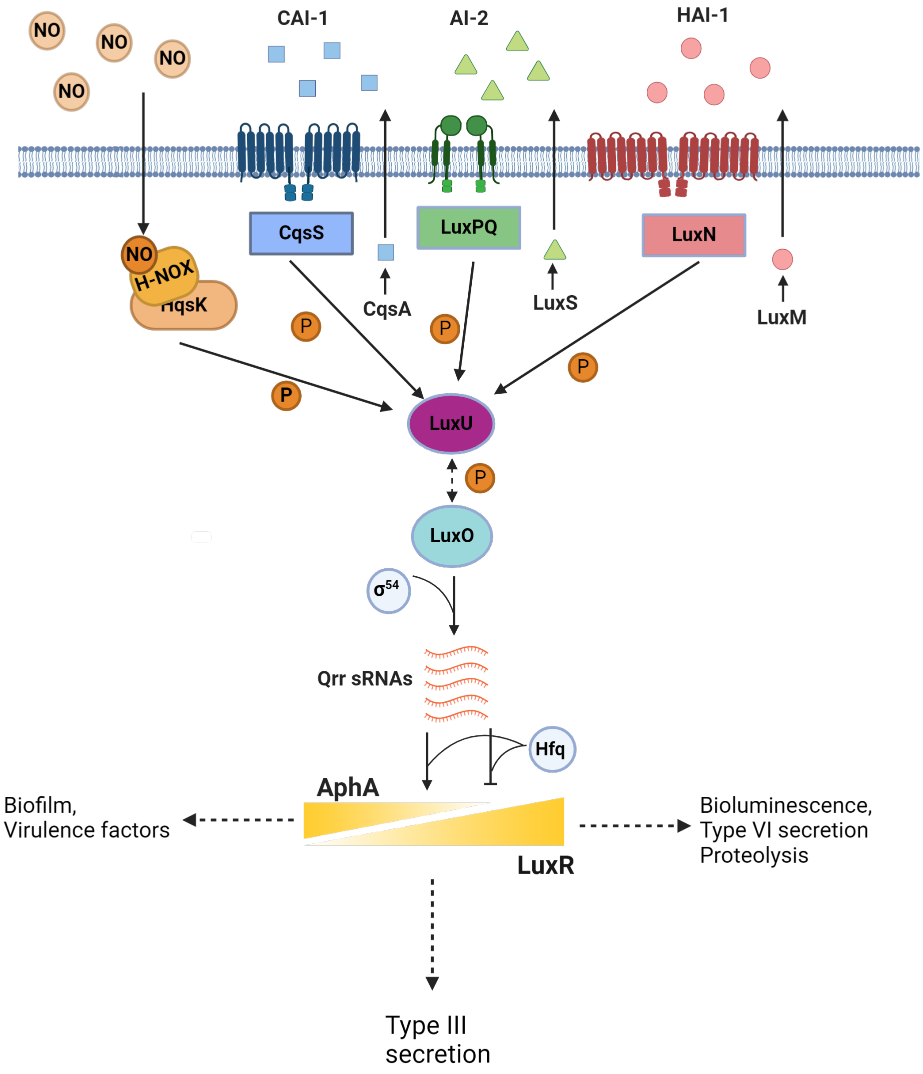

Figure S1. Schematic of the quorum sensing regulatory cascade in *V. campbellii* BB120. Membrane-bound receptors CqsS, LuxPQ, and LuxN and cytosolic HqsK act as kinases in the absence of autoinducers/nitric oxide (NO) at low cell densities, and phosphorylation of LuxU followed by LuxO enables LuxO~P to activate transcription of the Qrr sRNAs in concert with Hfq. The Qrrs degrade *luxR* mRNA and promote AphA translation, leading to activation of biofilm and virulence genes. At high cell densities, when autoinducers/nitric oxide are bound to their cognate receptors, the activity of these receptors switches to phosphatase, leading to loss of LuxO~P and no sRNA transcription. AphA ceases to be translated and LuxR is produced at high levels, leading to activation of bioluminescence, type VI secretion, and protease genes. Type III secretion genes are produced maximally when AphA is not present and LuxR is still at low levels at mid cell densities.

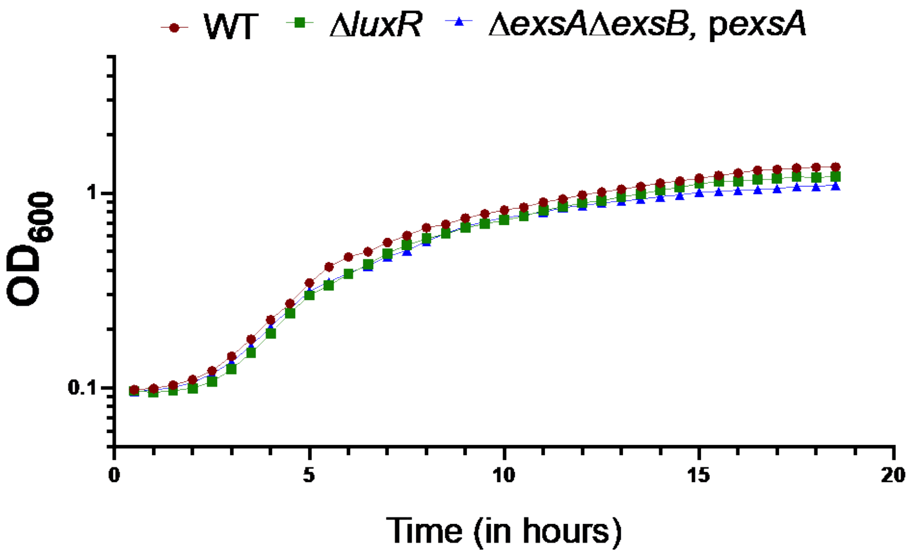

Figure S2. Growth curve analysis of isogenic *V. campbellii* strains. The strains include wildtype, Δ*luxR*, and Δ*exsA* Δ*exsB* with complementation of *exsA* from an ectopic plasmid (P_{tac}-$_{theo}$-*exsA*, pPP25). ExsA was induced using 10μM IPTG and 1mM theophylline in the strain Δ*exsA* Δ*exsB*, p*exsA* . The experiment was repeated in duplicate biological replicates. The growth curves were calculated over 18 hours of overnight growth.

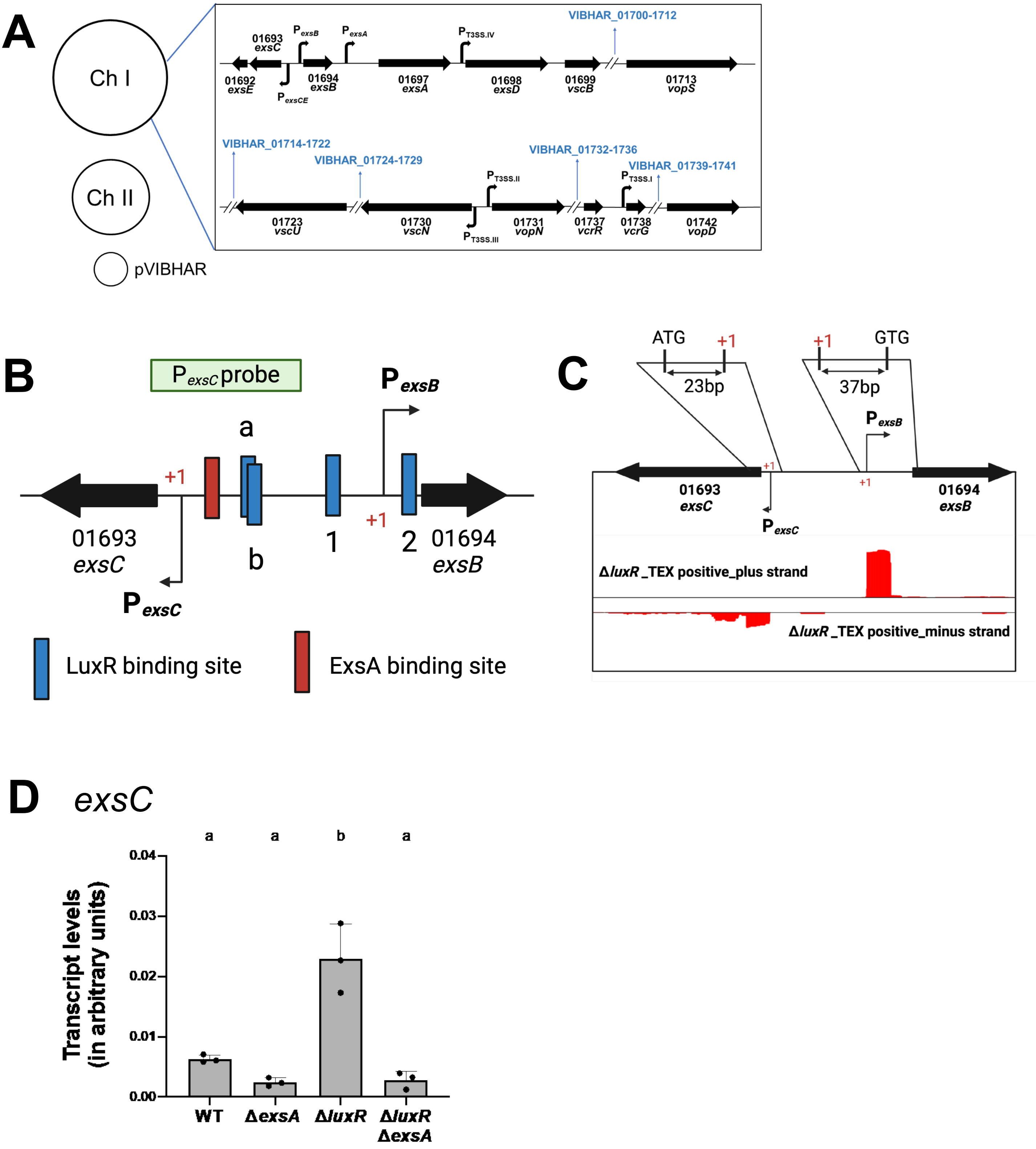

Figure S3. (A) Schematic of the arrangement of the known T3SS genes in a pathogenicity island and their further sub-grouping into four structural operons. The ~40 T3SS genes are arranged into a pathogenicity island on chromosome I and are further subdivided into four structural operons T3SS I-IV with promoters at *vcrG*, *vopN*, *vscN*, and *exsD,* respectively. ExsA activates transcription from the four promoters and thus activates the T3SS. (B) The schematic shows the LuxR and ExsA binding sites at the *exsC* and *exsB* promoters. The red boxes indicate ExsA binding site (BS), and the blue boxes indicate LuxR BSs a, b, 1, and 2. The gray boxes indicate the -10 and -35 sites. (C) Differential RNA sequencing analysis revealed the transcription start sites for the T3SS genes *exsBA* and *exsC*. (D) RT-qPCR measurements of *vopN* transcripts from cells collected at HCD (OD_{600} = 1.0) compared to internal control *hfq* gene for strains wild-type (WT), Δ*exsA*, Δ*luxR*, and Δ*exsA* Δ*luxR*.

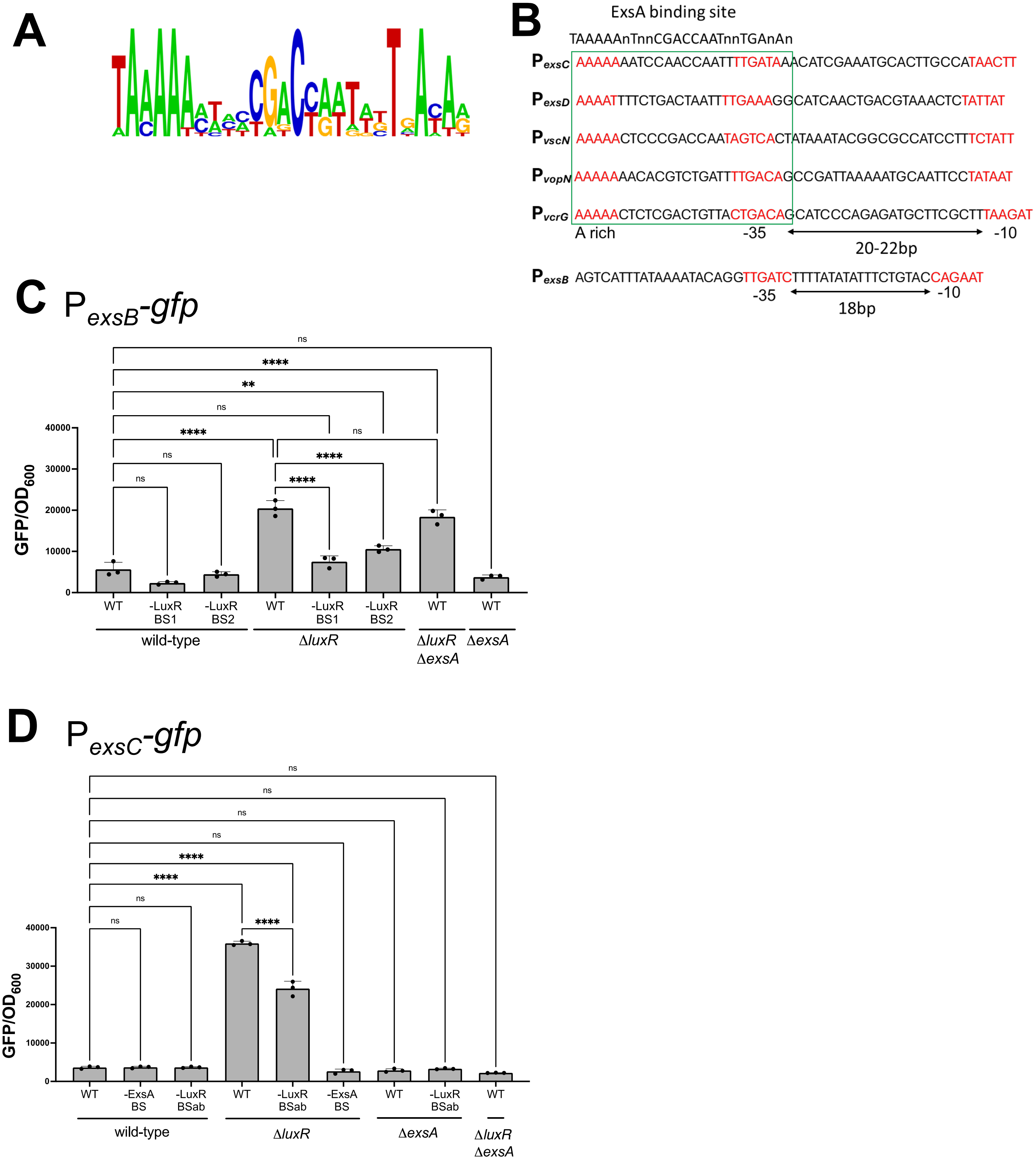

Figure S4. (A) The consensus site from *Pseudomonas aeruginosa* ExsA[44] was used to search the *V. campbellii* BB120 genome (qval at ≤0.01). 9 hits were identified and used to generate the putative BB120 ExsA position weight matrix (PWM) shown. (B) An *in silico* search for BB120 ExsA binding sites using the above PWM revealed putative ExsA binding sites at the *exsC*, *exsD*, *vscN*, *vopN*, and *vcrG* promoters. (C, D) GFP reporter assay results are shown for strains WT, Δ*exsA*, Δ*luxR*, and Δ*exsA* Δ*luxR* containing: (C) the P_{exsB}-*gfp* reporter plasmids (pPP07, pPP38, and pPP39) or (D) the P_{exsC}-*gfp* reporter plasmids (pPP51, pPP63, and pPP65), with either ExsA or LuxR binding sites (BS1, BS2) intact or deleted (denoted by "-") Error bars represent the standard deviation for triplicate biological replicates. A one-way analysis of variance (ANOVA) test was performed on normally distributed data (Shapiro-Wilk test) followed by Tukey's multiple comparisons test (p<0.05; n=3). Different letters indicate significant differences in pair-wise comparisons.

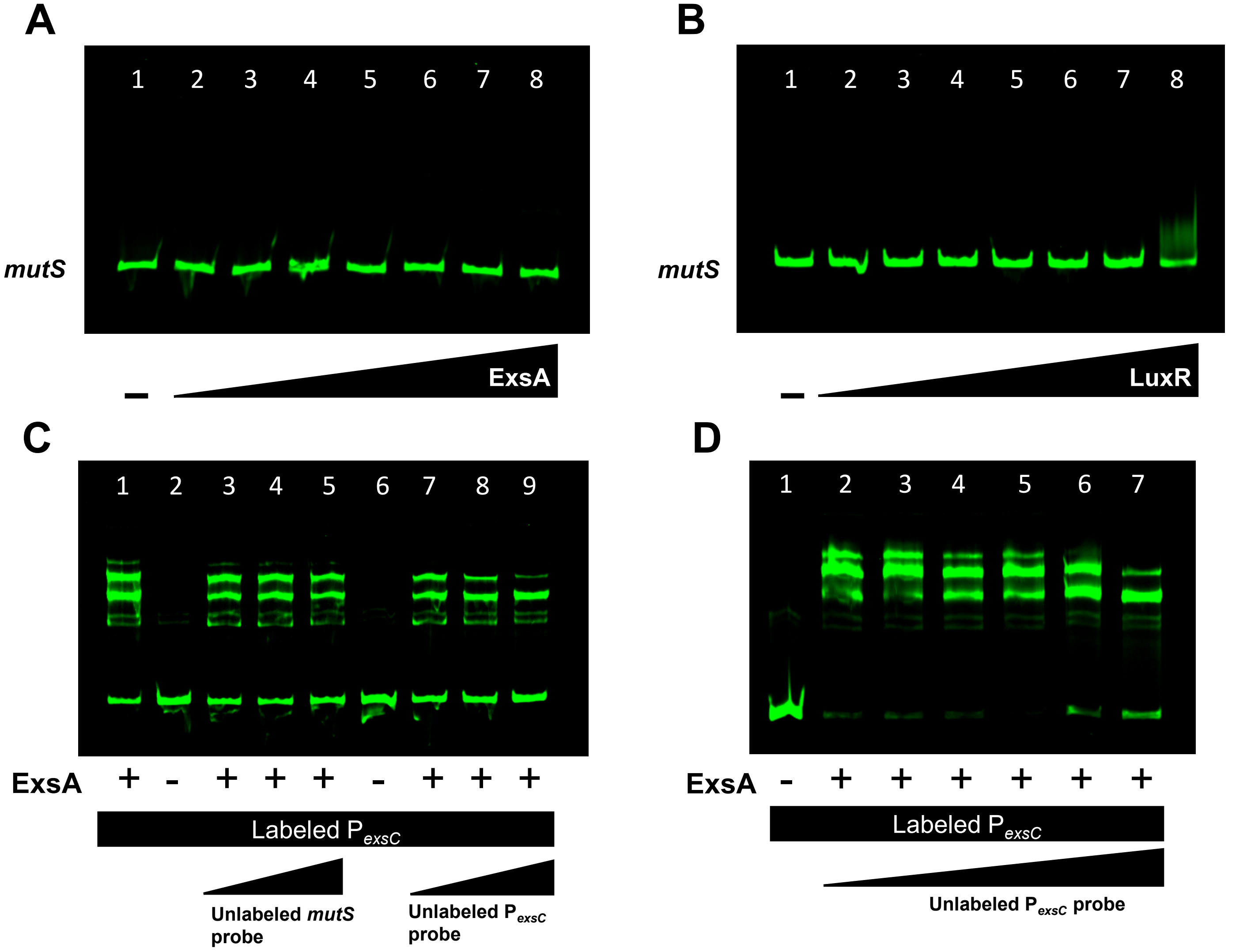

Figure S5. (A, B) EMSAs with 200-bp DNA probe (2 nM) corresponding to the *mutS* locus were assayed with: (A) Lanes 2-8: 4-fold dilution series of ExsA, diluted from 3 μM to 0.732 nM; or (B) Lanes 2-8: 5-fold dilution series of LuxR, diluted from 500 nM to 0.032 nM. Lane 1: no protein control. (C, D) EMSAs with fluorescently labeled P_{exsC} probe (2 nM) competed with either unlabeled P_{exsC} probe or unlabeled *mutS* probe. (C) Lane 1: 0.5 μM ExsA. Lanes 2 and 6: no protein control. Lanes 3-5: 0.5 μM ExsA competed with unlabeled *mutS* probe diluted in a 4-fold series from 100 nM to 6.25 nM. Lanes 7-9: 0.5 μM ExsA competed with unlabeled P_{exsC} probe diluted in a 4-fold dilution series from 100 nM to 6.25 nM. (D) Lane 1: no protein control. Lanes 2-7: 1 μM ExsA competed with unlabeled P_{exsC} probe diluted in a 4-fold dilution series from 100 nM to 0.098 nM concentrations.

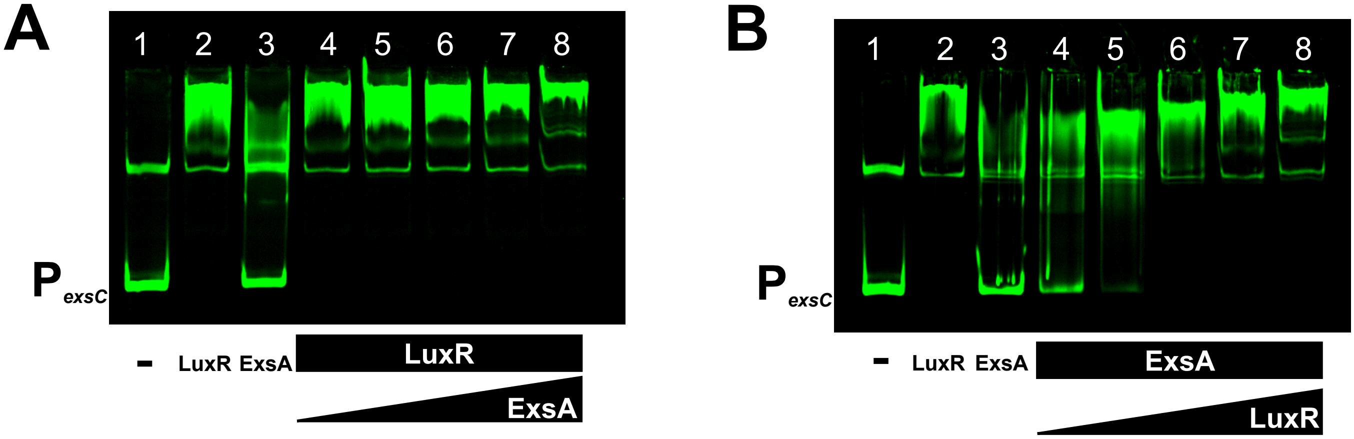

Figure S6. (A, B) Lane1, no protein control. Lane 2, LuxR alone at 250 nM concentration. Lane 3, ExsA alone at 500 nM concentration. Lanes 4-10, either LuxR or ExsA is added at a constant concentration of 250 nM or 500 nM respectively, followed by addition of either ExsA in a 2-fold dilution series from 500nM nM to 31.25 nM, or LuxR in a 2-fold dilution series from 250 nM to 15.625 nM.

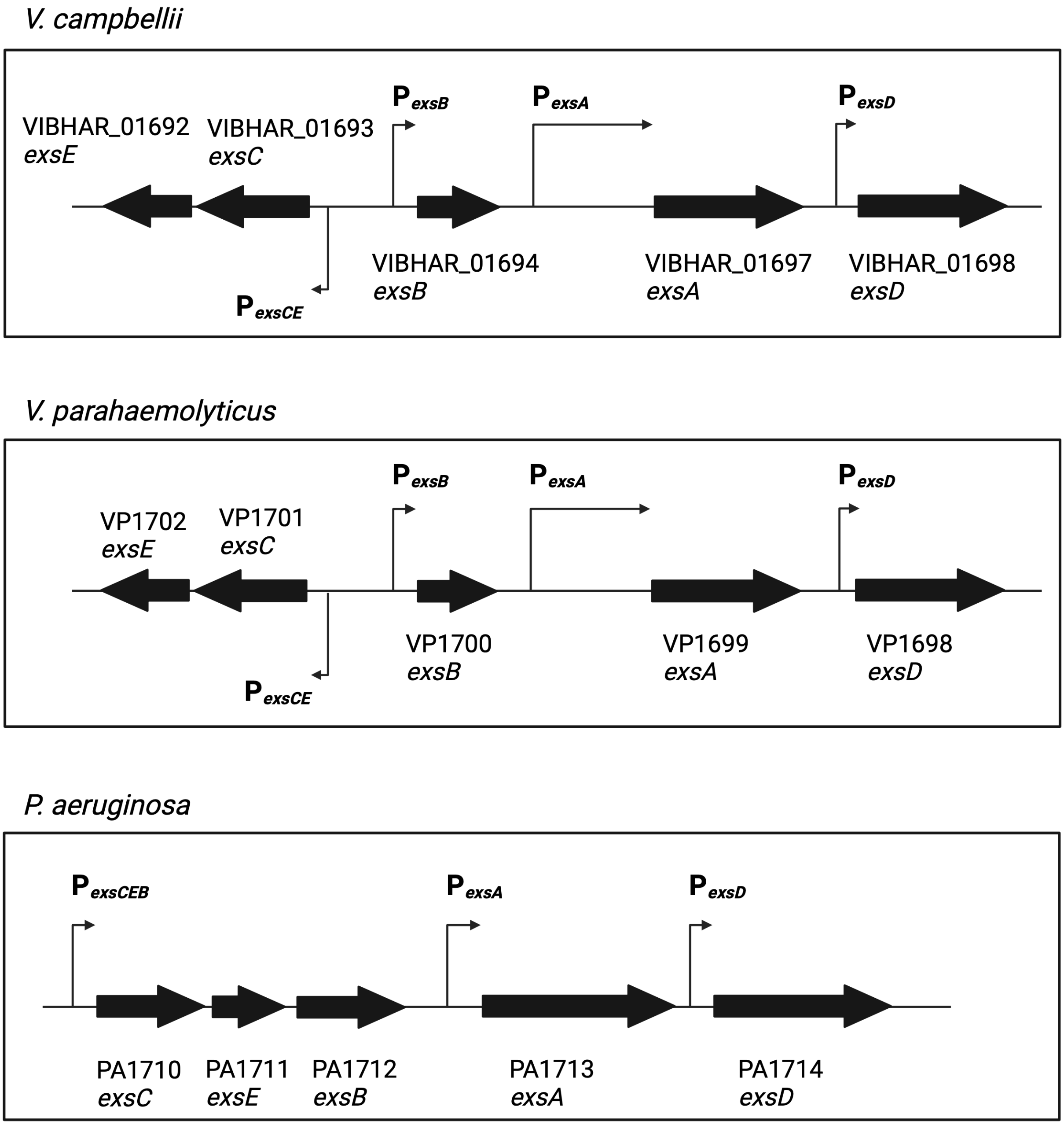

Figure S7. The schematic shows genetic organization of T3SS regulatory genes *exsA*, *exsB*, *exsC*, *exsE* and *exsD* in *V. campbellii* BB120, *V. parahaemolyticus* RIMD2210633, and *P. aeruginosa* PAO1.

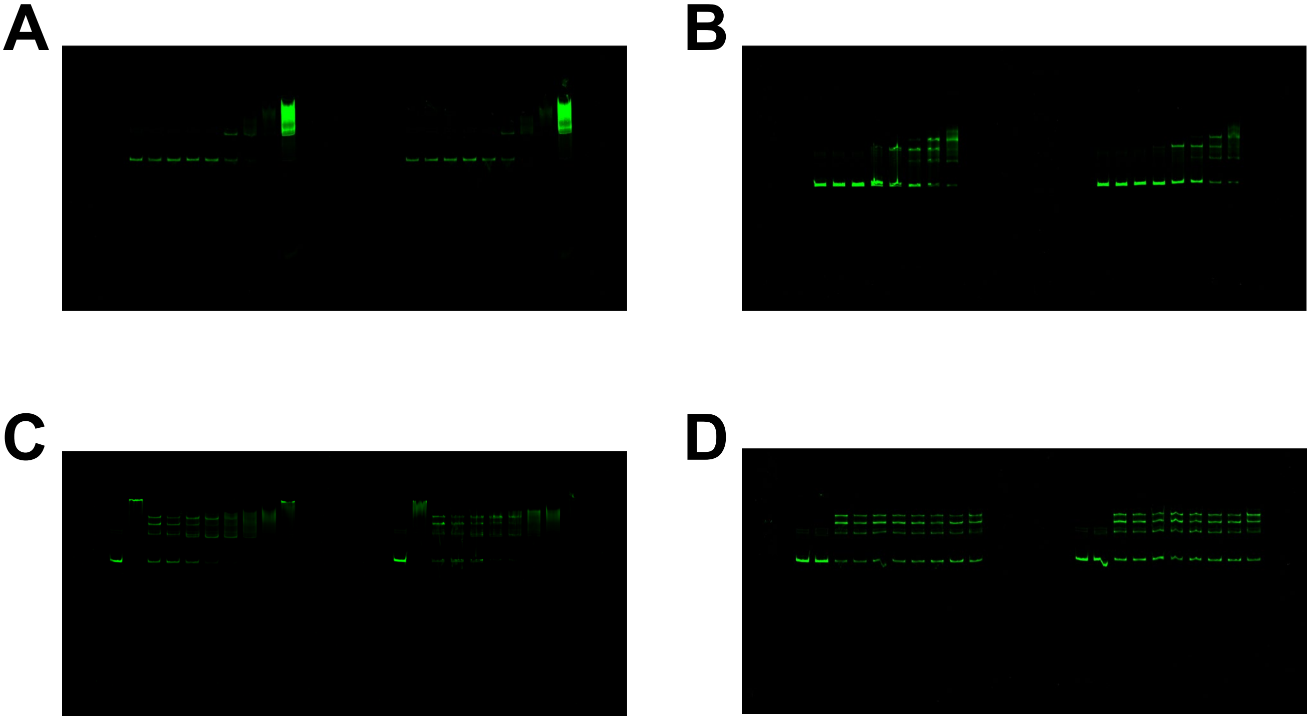

Figure S8. (A-D) Original, un-cropped, and un-edited electrophoretic mobility shift assay gel images for gels shown in figures 5A, 5B, 5C, and 5D, respectively.
